# TIMP3 expression associates with prognosis in colorectal cancer and its novel arylsulfonamide inducer, MPT0B390, inhibits tumor growth, metastasis and angiogenesis

**DOI:** 10.7150/thno.34020

**Published:** 2019-09-18

**Authors:** Han-Li Huang, Yi-Min Liu, Ting-Yi Sung, Tsui-Chin Huang, Ya-Wen Cheng, Jing-Ping Liou, Shiow-Lin Pan

**Affiliations:** 1 TMU Biomedical Commercialization Center, Taipei Medical University, Taipei 11031, Taiwan; 2 Ph.D Program in Biotechnology Research and Development, College of Pharmacy, Taipei Medical University, Taipei 11031, Taiwan.; 3 Graduate Institute of Cancer Molecular Biology and Drug Discovery, College of Medical Science and Technology, Taipei Medical University, Taipei 11031, Taiwan.; 4 School of Pharmacy, College of Pharmacy, Taipei Medical University, Taipei 11031, Taiwan.

**Keywords:** TIMP3, colorectal cancer, arylsulfonamide inducer, MPT0B390.

## Abstract

Tissue inhibitors of metalloproteinase 3 (TIMP3) are a major endogenous inhibitor of matrix metalloproteinase (MMPs) that inhibit tumor growth, invasion, metastasis and angiogenesis. In this study, we found that TIMP3 expression is associated with positive prognosis of colorectal cancer (CRC) clinicopathologically. Therefore, we developed a series of arylsulfonamide derivatives as *TIMP3* inducers in order to define potential colorectal cancer therapeutic agent. Among these, MPT0B390 was selected for anti-tumor, anti-metastasis, and anti-angiogenesis property determination.

**Methods:** The relationship between TIMP3 expression and clinical pathological features in colorectal patients and cell lines were determined by immunohistochemistry, bioinformatics analysis and western blotting. The anti-tumor function was validated by using MTT, apoptosis pathway detection and *in vivo* xenograft model for tumor growth inhibition determination. The anti-metastatic function was validated using a transwell migration assay, and using *in vivo* lung metastasis and liver metastasis models. The mechanism of MPT0B390-induced *TIMP3* expression was further tested using qPCR and Chromatin IP assay. The anti-angiogenesis function was examined by using transwell migration assay, and *in vivo* Matrigel plug assay.

**Results:** After screening candidate compounds, we identified MPT0B390 as an effective inducer of *TIMP3*. We showed that MPT0B390 induces *TIMP3* expression significantly and inhibits CRC cell growth *in vitro* and *in vivo*. By inducing TIMP3 expression, MPT0B390 can also exert its anti-metastasis effect to inhibit CRC cell migration and invasion and downregulates migration markers such as *uPA*, *uPAR*, and *c-Met*. Subsequent Chromatin immunoprecipitation assay revealed that MPT0B390 can significantly inhibit EZH2 expression as well as its binding to *TIMP3* promoter region to regulate *TIMP3* induction. In addition to the anti-tumor and anti-metastasis capability, MPT0B390 can also induce *TIMP3* expression in endothelial cells to inhibit tumor angiogenesis.

**Conclusion:** These data suggest the potential therapeutic applications of the *TIMP3* inducer, MPT0B390, for colorectal cancer treatment.

## Introduction

Colorectal cancer (CRC) is not only the third most common malignancy, but one of the leading cause of deaths in the world 1. Late-stage treatment includes combined chemotherapy with target therapies such as bevacizumab and cetuximab to inhibit tumor growth and angiogenesis 2. However, the response rate remains unsatisfactory without predictable clinical outcome that could be easily monitored. Therefore, new therapeutic agents with target molecules associated with clinical and pathological features will greatly benefit the treatment.

The tissue inhibitors of metalloproteinases (TIMPs) are tissue specific and have various modes of regulation. Four mammalian TIMPs have been characterized to contained basic similarities, but distinct structural features, biochemical properties and expression patterns 3. TIMP3, an extracellular matrix (ECM) protein unlike other TIMP family members, is sequestered to the ECM by binding to heparan sulphate proteoglycan and is a potential inhibitor of some membrane-associated ADAMs 4. High expression of TIMP3 has been found to promote apoptosis in various tumor types *in vitro* and *in vivo* 5-8. Besides, TIMP3 has been reported to regulate endothelial apoptosis and direct interaction with vascular endothelial growth factor (VEGF) receptor-2 to inhibit angiogenesis and tumor progression 9-11, or as a tumor suppressor by modulating tumor migration, invasion, and tumorigenicity 6, 12. These evidences suggest that overexpression of TIMP3 is a rational multi-phenotypic approach for cancer treatment. Indeed, studies have shown that adenovirally delivered TIMP3 overexpression reduced blood vessel density, promoted apoptosis and significantly reduced tumor growth in melanoma *in vivo* model 5 and suppressed malignant behaviors such as migration, invasion and tumor growth of colorectal cancer cells 13. Therefore, TIMP3 may be exploited as a potential target for cancer treatment with various therapeutic benefits.

**Figure 1:**
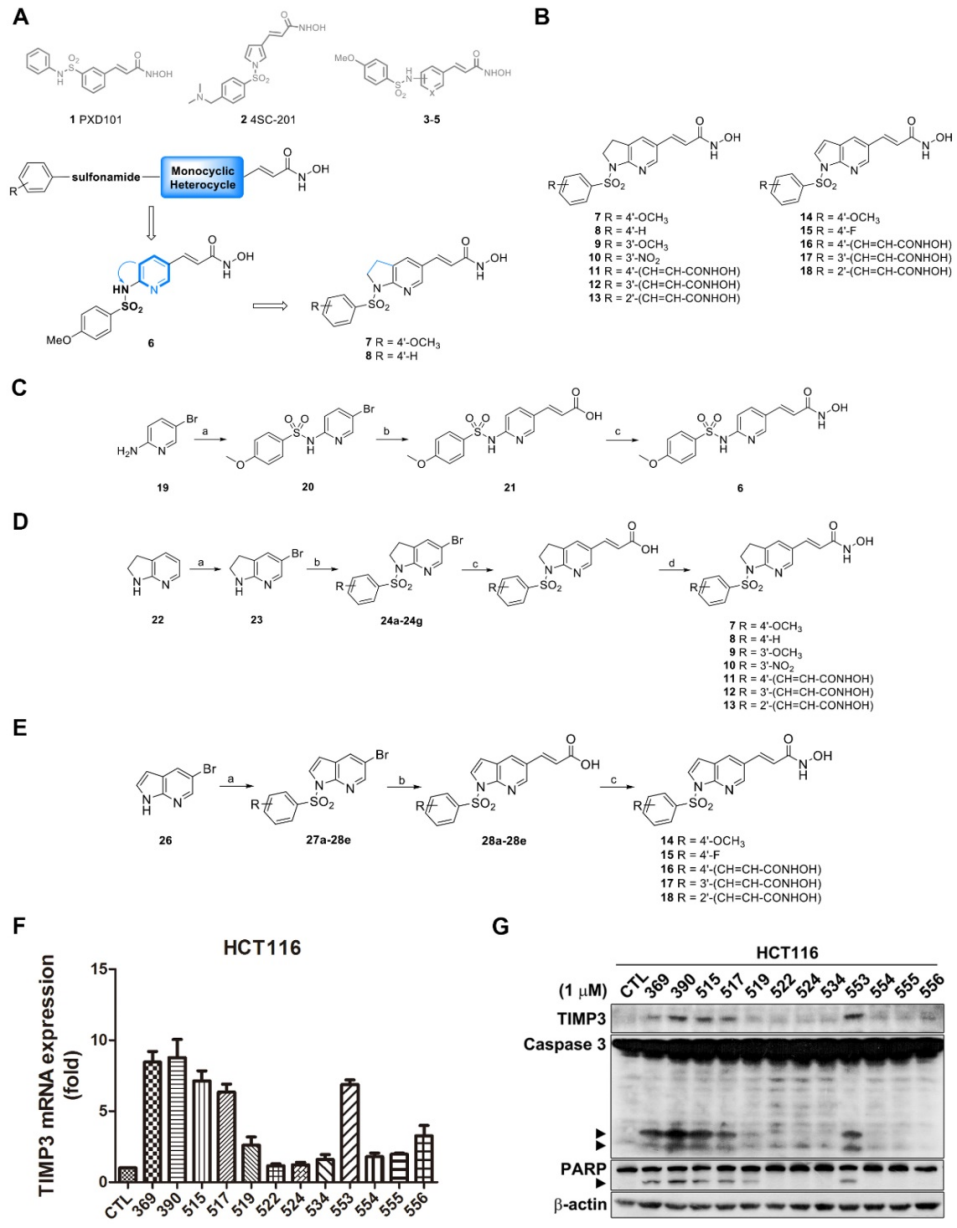
** Synthesis route and TIMP3 induction ability of TIMP3 activators.** (A) Proposed desired target compounds (**6**-**8**). (B) Proposed desired target compounds (**7**-**18**). (C) Reagents and conditions: (a) 4-methoxybenzenesulfonyl chloride, DMAP, ACN, pyridine, rt; (b) (i) Pd(OAc)_2_, triphenylphosphine, Et_3_N, NaHCO_3_, DMF, *tert*-butyl acrylate, 120 ^o^C; (ii) 1M LiOH_(aq)_, dioxane, 40 ^o^C (c) (i) NH_2_OTHP, EDC‧HCl, HOBt, NMM, DMF, rt.; (ii) 10% TFA_(aq)_, CH_3_OH, rt. (D) Reagents and conditions: (a) bromine, pyridine, CH_2_Cl_2_, rt; (b) substituted arylbenzenesulfonyl chlorides, pyridine, reflux; (c) (i) Pd(OAc)_2_, triphenylphosphine, Et_3_N, NaHCO_3_, DMF, methyl acrylate, 120 ^o^C; (ii) 1M LiOH_(aq)_, dioxane, 40 ^o^C or (i) Pd(OAc)_2_, triphenylphosphine, Et_3_N, NaHCO_3_, DMF, *t*-Butyl acrylate, 120 ^o^C; (ii) TFA, rt; (d) (i) NH_2_OTHP, EDC‧HCl, HOBt, NMM, DMF, rt; (ii) 10% TFA_(aq)_, CH_3_OH, rt. (E) Reagents and conditions: (a) substituted arylbenzenesulfonyl chlorides, pyridine, reflux; (b) (i) Pd(OAc)_2_, triphenylphosphine, Et_3_N, NaHCO_3_, DMF, *t*-Butyl acrylate, 120 ^o^C; (ii) TFA, rt; (c) (i) NH_2_OTHP, EDC‧HCl, HOBt, NMM, DMF, rt; (ii) 10% TFA_(aq)_, CH_3_OH, rt. (F) TIMP3 mRNA level induced by sulfonamide derivatives. HCT116 cells were treated with indicated compounds for 24 h and mRNA were extracted and measured by real-time PCR. Data are expressed as mean ± SEM of at least three independent experiments. (G) TIMP3 expression elevated by sulfonamide derivatives. HCT116 cells were treated with indicated compounds for 48 h. Cell lysates were subjected to western blot for protein detection.

In our previous findings, we investigated the arylsulfonamide derivative MPT0G013 as a potent inhibitor of antiangiogenic activities by activating 14, 15. The literature survey indicates that hydroxamic acid contributes to the histone deacetylase (HDAC) inhibition activity through interfering with the binding mode of zinc ion at the catalytic site, and it becomes the symbolic moiety of HDAC inhibitors 16-18. Scientific attentions are therefore comprehensively drawn to the auxiliary linker section and recognition area that increases the structural diversity. Our previous works utilized indole and indoline as a linker connecting to the benzenesulfonamide cap 15, 19. The promising results encouraged us to explore the linker effect on HDAC inhibitory activity while maintaining the benzenesulfonamide moiety. Screening various potent HDAC inhibitors such as PXD101 (**1**, Belinostat, Approved) and 4SC-201 (**2**, Resminostat, Phase II), similar structural alignment was observed, aryl rings-sulfonamide bond-monocyclic heterocycle-*N*-hydroxyacrylaminde (Figure 1A). On the basis of the published papers, N-hydroxyphenylacrylamides (**3**), N-hydroxypyridinylacrylamides (**4**) and “reverse” sulfonamide derivatives (**5**) as the core structures have been described. All of the above compounds have shown potency for HDAC inhibition 20, 21.

In the beginning, the monocyclic pyridine was embedded in the structure to afford compound **6** on the basis of the above symbolic alignments. The preliminary result indicated that pyridine is detrimental to biological activity (data not shown). The advanced modification of compound **6** under ring-expansion yielded compounds **7** and **8** which contain a bicyclic 7-azaindoline (Figure 1A). Notably, compounds **7** and **8** exhibited marked biological activity as compared to **6** (Table 1). As a result, a series of 7-azaindoline (**7-13**) and 7-azaindole (**14**-**18**) analogues has been synthesized from using the heterocycles as a core structure to connect with the substituted sulfonamide groups and allowed evaluation of bioactivity and served as potent HDAC inhibitors in this study (Figure 1B).

Herein, we found that TIMP3 expression correlated clinical pathologically with colorectal cancer patient, which urged us to develop *TIMP3* inducers as therapeutic agents for colorectal cancer treatment. After screening series of arylsulfonamide derivatives, we found that MPT0B390 (3-[1-(3,4-dimethoxy-benzenesulfonyl)-1H-indol-5-yl]-N-hydroxy-acrylamide) can significantly upregulate TIMP3 expression in colorectal cancer cells and exhibit potent anti-tumor, anti-metastasis and anti-angiogenic effect *in vivo*, which indicates the potential usage of MPT0B390 as a therapeutic agent with multiple inhibitory aspects against colorectal cancer growth and angiogenesis.

## Materials and methods

### Study subjects

Primary colorectal cancer patients were admitted to Taipei Medical University Hospital. All patients wrote informed consent approved by the Institutional Review Board (IRB number:106IRB0417). Before surgical therapy, the enrolled patients did not receive any chemotherapy or radiation therapy. The TNM stages of all colorectal cancer patients were determined according to the American Joint Committee on Cancer/International Union Against Cancer TNM staging system.

### Chemistry and synthesis of N-1 sulfonamides 6,5 membered ring with N-hydroxyacrylamides (1-arylsulfonyl-5-(N-hydroxyacrylamide)azaindolines)

Nuclear magnetic resonance (^1^H NMR) spectra were obtained with Bruker DRX-500 spectrometer (operating at 500 MHz) and Bruker Fourier 300 (operating at 300 MHz), with chemical shift in parts per million (ppm, *δ*) downfield from TMS as an internal standard. High-resolution mass spectra (HRMS) were measured with a Finnigan Mat 95S electronspray ionization (ESI) mass spectrometer. Purity of the final compounds were determined using a Hitachi 2000 series HPLC system using C-18 column (Agilent ZORBAX Eclipse XDB-C18 5 μm. 4.6 mm × 150 mm) and were found to be ≥ 95%. Flash column chromatography was done using silica gel (Merck Kieselgel 60, No. 9385, 230-400 mesh ASTM). All reactions were carried out under an atmosphere of dry nitrogen. The detailed synthesis routes of each compounds were described in Supporting Information.

### Cell culture and reagents

Colorectal cancer cells HCT116, HT29, and Colo205, human umbilical vein/vascular endothelium cell HUVEC and mouse fibroblast cell NIH3T3 were purchased from American Type Culture Collection (Manassas, VA, USA). Human colon epithelial cells FHC, colorectal cancer cells SW480 and SW620 were kind gifts from Dr. Ya-Wen Cheng (The Ph.D. Program for Cancer Biology and Drug Discovery, College of Medical Science and Technology, Taipei Medical University). Human colorectal cancer cells HCT15 and DLD-1 were kind gifts from Dr. Tsui-Chin Huang (The Ph.D. Program for Cancer Biology and Drug Discovery, College of Medical Science and Technology, Taipei Medical University). Human colorectal cancer cells Caco2 was a kind gift from Dr. Che-Ming Teng (Pharmacological Institute, College of Medicine, National Taiwan University). HUVEC cells were maintained in M199 (supplement with 20% FBS (v/v), 1 mM sodium pyruvate, 2 mM L-glutamine and 5000 I.U. heparin). FHC cells were cultured in DMEM/F12 (supplement with 25 mM, 10 ng/ml cholera toxin, 0.10 μg/ml insulin, 0.005 mg/ml transferrin, 0.5 μg/ml hydrocortisone, 20 ng/ml EGF, 1 mM sodium pyruvate and 1% NEAA). SW480 and SW620 cells were cultured in L-15 (supplement with 1 mM sodium pyruvate and 1% NEAA). HCT116, HT29, DLD-1 and HCT15 were cultured in RPMI-1640. Caco-2 cells were cultured in DMEM. Except M199 supplemented with 20 % FBS, all other medium contained 10% FBS and penicillin (100 units/ml)/streptomycin (100 μg/ml)/amphotericin B (0.25 μg/ml). All cells were maintained in humidified air containing 5% CO_2_ at 37°C and cultured every 2-3 days.

MPT0B390 and derivatives were synthesized by Professor Jing-Ping Liou (Taipei Medical University). Tissue culture medium, Fetal Bovine Serum (FBS), penicillin, streptomycin, and trypsin were obtained from GIBCO/BRL Life Technologies (Grand Island, NY, USA). 3-(4,5-Dimethylthiazol-2-yl)-2,5-diphenyltetrazolium bromide (MTT) and all of the other chemical reagents were purchased from Sigma Chemical (St. Louis, MO, USA). The following antibodies were used: EZH2, H3K27Me3 (Cell Signaling Technologies, Boston, MA, USA); PARP, HRP-conjugated anti-mouse and anti-rabbit IgG (Santa Cruz Biotechnology, Santa Cruz, CA, USA); caspase 3 (Imgenex, San Diego, CA, USA); TIMP3, Actin (Millipore, Billerica, MA, USA). Trizol reagent was from Invitrogen (Carlsbad, CA, USA). Random primer and moloney murine leukemia virus reverse transcriptase (M-MLVRT) were purchased from Promega (Madison, WI, USA). SYBR green mastermix was from Applied Biosystems (Carlsbad, California, USA).

### Sulforhodamine B (SRB) assay

HCT-116 cells were seeded in 96-well plates overnight. Basal cells were fixed with 10% trichloroacetic acid (TCA) representing cell population at the time of drug treatment. After incubation with vehicle or test compounds for 48 h, cells were then fixed with 10% TCA and stained with SRB at 0.4% (w/v) in 1% acetic acid. Excess SRB was washed away by 1% acetic acid and stained cells were lysed with 10mM Trizma base. The absorbance was measured at wavelength of 515 nm. Growth inhibition of 50% (GI_50_) is determined at the drug concentration that results in 50% reduction of total protein increased in control cells compare to basal cells during compound incubation.

### MTT assay

Cell viability was assessed by mitochondria dehydrogenases activity, forming an insoluble blue formazan product after reducing the tetrazolium ring of MTT. HCT116 and HT29 cells were plated in 96-well plate (5000 cells/well) and treated with 200 µl of indicated agents at different dosages for 48 h. After treatment, 100 µl of 0.5 mg/ml MTT were added to each well and incubated at 37°C for 1 h. MTT-containing medium were removed and 100 µl DMSO were added to each well to lyse cells. Plates were then measured at 550 nm using an enzyme-linked immunosorbent assay (ELISA) reader (Packard, Meriden, CT, USA).

### Lentiviral transfection and infection

Lentivirial plasmids of target genes were obtained from Academia Sinica (Taipei, Taiwan) and listed as following: pLAS.VOID (negative control), shTIMP3 (#2413, #2414). Lentivirial plasmids were cotransfected with the 3^rd^ Generation Packaging Systems (pMDLg/pRRE, #12251; pRSV-Rev, #12253; pMD2.G #12259) (Addgene; Cambridge, MA) in 293T cells using Lipofectamine 2000 (Thermo Fisher Scientific) according to manufacturers' instructions. Viral particles were collected to infect target cells followed by puromycin selection for one week to generate stable cell lines.

### RNA extraction and real-time PCR

Total RNA was isolated using TRIzol and Direct-zol™ RNA MiniPrep kit (ZYMO research; Irvine, CA, USA) followed manufacturer's instruction. Reverse-transcription to cDNA was performed using random primer and M-MLRT. In brief, first strand cDNA was synthesized using 1 µg of mRNA incubating with random primer at 65°C for 5 min and then reacting with M-MLRT at 37°C for 1 h. For real-time PCR, cDNAs were amplified in SYBR Green PCR Master Mix (Life Technologies; Grand Island, NY, USA) and detected with the Applied Biosystems StepOnePlus™ Q-PCR detection system. Relative gene expression was normalized to GAPDH and calculated by using the 2(^-∆∆CT^) method.

### HDAC activity assays

The IC_50_ values of HDAC inhibitors were determined by carrying out a fluorimetric histone deacetylase assay following the manufacturer's instructions. Histone deacetylase isomer inhibition assays were done by using purified recombinant histone deacetylase proteins of the various isomers (BPS Bioscience Inc.). Reactions were prepared in 0.1 mol/L KCl, 20 mmol/L HEPES/NaOH at pH 7.9, 20% glycerol, 0.2 mmol/L DTA, 0.5 mmol/L DTT, and 0.5 mmol/L phenylmethylsulfonylfluoride. The HDAC assay was done by using Fluor-de-Lys substrate and concentrations (nmol/L to μmol/L) of compounds at 37 °C in HDAC assay buffer, containing 25 mmol/L Tris-Cl, pH 8.0, 137 mmol/L NaCl, 2.7 mmol/L KCl, and 1 mmol/L MgCl_2_. Fluorescence was measured with excitation at a wavelength of 360 nm and emitted light of 460 nm was detected by SpectraMax M5 Multi-Mode Microplate Reader. Negative (no enzyme, no inhibitor, a drug with no HDAC inhibition activity) and positive controls (with no HDAC inhibitor and known HDAC inhibitor SAHA) were included in the assay reactions. Half maximal inhibitory concentration (IC_50_) is determined at the drug concentration that results in 50% reduction of HDAC activity increase in control wells during the compound incubation. The reaction was done in triplicate for each sample.

### Protein extraction and western blot

Cells were treated with indicated condition and then harvested in lysis buffer (20 mM Tris-HCl pH 7.5, 150 mM NaCl, 1 mM Na_2_EDTA, 1 mM EGTA, 1% NP-40, 1% Sodium Deoxycholate, 0.1% SDS, 2.5 mM β-glycerolphosphate, 1 mM Na_4_P_2_O_7_, 5 mM NaF, 1 mM Na_3_VO_4_ and protease inhibitor cocktail from Millipore) incubated on ice for 30 min followed by centrifugation at 13000 rpm for 30 min. Total protein was determined and equal amounts of protein were separated by 8-15% sodium dodecyl sulfate-polyacrylamide gel electrophoresis (SDS-PAGE) and transferred to poly(vinylidene difluoride) (PVDF) membranes. Membranes were immunoblotted with specific antibodies overnight at 4°C and then applied to appropriate horseradish peroxidase-conjugated anti-mouse or anti-rabbit IgG secondary antibodies for 1 h at room temperature. Signals were visualized using an enhanced chemiluminescence (Amersham, Buckinghamshire, UK).

### Migration assay

Assay was performed in 6.5 mm transwell with 8.0 µm pore polycarbonate membrane insert in a 24-well format (Corning Inc.; Corning, NY, USA). HCT116 cells were seeded into upper chambers at 1 × 10^5^ cells in serum free-medium and incubated with indicated condition for 24 h for cell migration. HUVEC cells were pretreated with indicated condition for 24 h and then seeded into upper chambers at 1 × 10^5^ cells in EBM2 basal medium and incubated with indicated condition for 6 h for migration. Membranes then fixed with 10% neutral buffered formalin at room temperature for 10 min and stained with crystal violet (Sigma-Aldrich; St. Louis, MO) at room temperature for 20 min. Membranes were washed with water twice and no-migrated cells were scrap off by cotton stick. Crystal violet of migrated cells was dissolved using 33% acetic acid and then detected at wavelength 600 nm.

### Transient transfection

Silencer select siRNA against human *TIMP3* was purchased from Ambion (Austin, TX, USA). Human EZH2 siRNA Smartpool was purchased from Dharmacon (Lafayette, CO, USA). Colon cancer cells were transfected with Lipofectamine RNAiMAX Transfection Reagent (Invitrogen, Carlsbad, CA, USA) according to the manufacturer's instruction. After transfection, cells were recovered for 24 h incubating at 37°C and then harvested for real-time PCR assay and Western blot analysis.

### Chromatin immunoprecipitation (ChIP)

Chromatin immunoprecipitation was performed using EZ-Magna ChIP A/G kit (Millipore, Billerica, MA, USA) according to manufacturer's instruction. Briefly, 1.2 × 10^6^ HCT116 cells were seeded in 10-cm dish followed by indicated treatment for 24 h. Cells were then cross-linked in 1 % formaldehyde and quenched in 0.125 M glycine. Cells were lysed and nuclear fraction were sonicated to get sheared DNA. Equivalent crosslinked protein/DNA were immunoprecipitated with protein A/G magnetic beads and specific antibodies at 4 °C overnight. Beads were then washed sequentially with the low-salt, high-salt, LiCl, and TE buffers. After elution of the protein-DNA complexes, proteinase K was added and incubated at 62 °C for 2 h with shaking to reverse the cross-links to DNA fragments. Free DNA were purified and analyzed by real-time PCR. Specific ChIP primer sequences were listed in the Supplementary Table S3.

### *In vivo* animal model

All animal experiments used in this study followed ethical standards, and protocols have been reviewed and approved by Animal Use and Management Committee of Taipei Medical University (IACUC approved No. TMU-LAC-2015-0113).

For *in vivo* xenograft model, male nude mice of 9-week old were injected subcutaneously with the same volume of BD Matrigel Matrix HC (catalog 354248, BD bioscience), and HCT116 cells (2.6×10^6^ cell/mouse) into the flank of each animal. When the tumors had grown to around 100 mm^3^, animals were divided into three groups (n=6) and receive the following treatment by oral gavage for 18 days during the study: (a) vehicle alone, (b) MPT0B390 at 10 mg/kg daily, and (c) MPT0B390 at 25 mg/kg daily. MPT0B390 was dissolved in vehicle [1% carboxymethyl cellulose (CMC) + 0.5% Tween 80]. Tumor size was measured twice weekly and calculated from V = l*w^2^/2, where w = width (w) and l = length (l).

For *in vivo* lung metastasis assay, 5-week old male balb/c mice were used and divided into two groups with 4 animals in each group. 1×10^5^ CT-26 mouse colorectal cancer cells were injected intravenously into the lateral tail vein and receive the following treatment by oral gavage during the study: (a) vehicle alone, and (b) MPT0B390 at 50 mg/kg daily. After 3 weeks of treatment, mice were sacrificed and lung tissues were dissected, weighed, and photographed. For *in vivo* liver metastasis assay, 6-week old male nude mice were used and divided into three groups (n=3-6): (a) sham, (b) control, and (c) MPT0B390 at 25 mg/kg daily. 6.78×10^6^ HCT-116 human colorectal cancer cells were injected into the spleen. Mice were received vehicle or MPT0B390 by oral gavage during the study. After three weeks of inoculation, mice were euthanized and liver was collected, weighed, fixed in 10% neutral formalin and then photographed.

For *in vivo* matrigel plug assay, male nude mice were used and divided into four groups (basal, n=2; control, n=5, MPT0B390 1 μM, n=3; MPT0B390 10 μM, n=3). Mice were subcutaneously injected with 100 μl Matrigel (BD Bioscience) mixed with angiogenic factors (VEGF, bFGF, IGF-1, and EGF, 40 ng/ml of each) and with or without MPT0B390 (1 μM or 10 μM). After seven days, the animals were sacrificed and carefully dissected for photographing. To quantify the blood vessel formation, hemoglobin content was analyzed by Drabkin's reagent kit (Sigma Chemical; St. Louis, MO, USA).

### Statistical analysis

Student's t-test was used to compare the mean of each group with that of the control group in experiments and one-way ANOVA was used in animal study. P-values less than 0.05 were considered significant (*P<0.05, **P<0.01, ***P<0.001).

## Results

### TIMP3 expression is elevated in tumor tissue and negatively associates with prognosis in colon cancer

**Figure 2:**
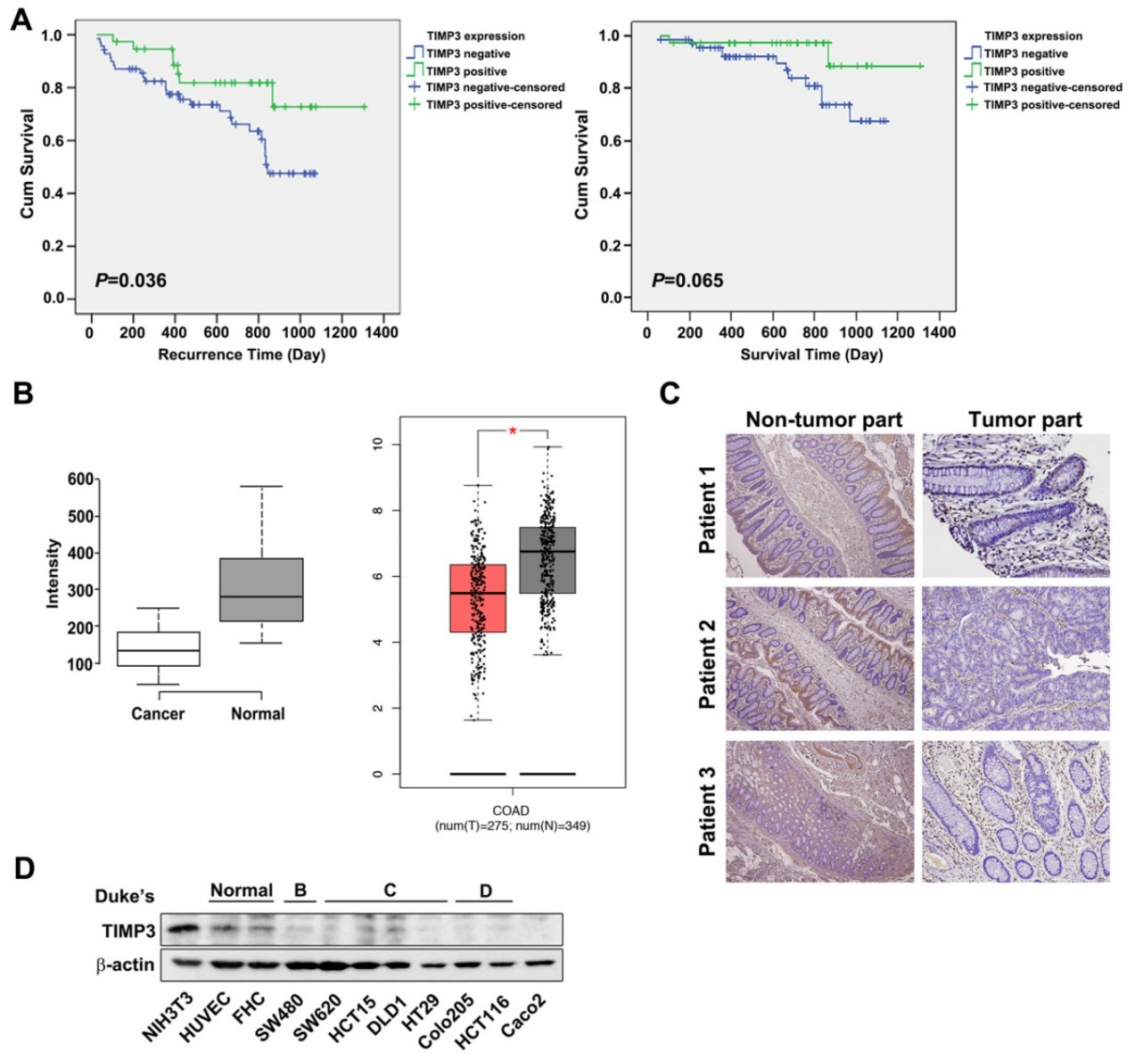
** TIMP3 exhibited positive correlation with disease stages and recurrence time in colon cancer.** (A) Kaplan-Meier analysis for the influence of TIMP-3 on recurrence time and survival time. (B) Expression level of TIMP3 in colorectal cancer (GSE8671, n = 32, p = 6.7 x 10-9, *left panel*; GEPIA expression database, n = 257 in cancerous tissue sand n = 159 in normal tissue, *right panel*). Center lines show the medians; box limits indicate the 25^th^ and 75^th^ percentiles as determined by R software; whiskers extend 1.5 times the interquartile range from the 25th and 75th percentiles, outliers are represented by dots. Two-tailed Student's t test was used to determine all P values. (C) TIMP3 expression of three CRC patients in non-tumor part and tumor part tissue. (D) Different colorectal cancer cells were collected followed by western blot for TIMP3 detection. NIH3T3 were loaded as positive control. HUVEC and FHC cells were loaded as normal cells for comparison.

TIMP3 has been reported to be downregulated in various cancer types such as thyroid cancer, due to aberrant promoter hypermethylation 12, 22 and plays a role in tumor migration, invasion and angiogenesis 11, 12. To characterize the relationships between TIMP3 expression and clinical and pathological features in CRC, we examined 191 samples from CRC patients (Table 2). The representative TIMP3 expression score was shown in Supplementary Figure S1. According to our results, TIMP3 expression is statistically correlated with the distant metastasis (M factor), and the four stages of disease progression (Table 2). Kaplan-Meier analysis showed that TIMP3 expression was negatively associated with recurrence time (*P*=0.036). Patients with higher TIMP3 expression had longer recurrence time than those with lower TIMP3 expression (Figure 2A, *left panel*). However, the survival time in patients with higher TIMP3 expression showed a similar trend, but was not statistically significant (*P*=0.065, Figure 2A, *right panel*). *TIMP3* expression also exhibited higher expression in normal tissues compared to cancerous region using bioinformatics analysis from the Gene Expression Omnibus (GEO) database (Figure 2 B, *left panel*), Gene Expression Profiling Interactive Analysis (GEPIA) (Figure 2B,) database (Figure 2B,* right panel*), and immunohistochemical staining in three clinical CRC patients' tissues (Figure 2C). Based on Duke's classification of colorectal cancer cell lines, TIMP3 is expressed abundantly in normal cells, whereas malignant CRC cells are correlated to disease stage progression (Figure 2D). Taken together, these data suggest that higher TIMP3 expression is negatively associated with clinical CRC prognosis. Therefore, TIMP3 shows potential as a clinically relevant target for cancer treatment, which prompted us to further synthesize *TIMP3* inducers as potential therapeutic agents against CRC.

### Synthesis and screening of N-1 sulfonamides 6,5 membered ring with N-hydroxyacrylamides (1-arylsulfonyl-5-(N-hydroxyacrylamide)azaindolines), which transcriptionally activate TIMP3 expression and lead to CRC cells apoptosis, as potential TIMP3 activators

According to our previous work, sulfonamide-based HDAC inhibitors increased TIMP3 expression to inhibit tumor angiogenesis 14. Therefore, we designed and synthesized a series of sulfonamide compounds as potential *TIMP3* inducers. Based on the ring-expansion concept and the structure of current HDAC inhibitors, a series of 7-azaindoline (**7**-**13**) and 7-azaindole (**14**-**18**) analogues have been synthesized (Figure 1B). All of the above compounds are proved the potency for HDAC inhibition (Table 1 and Supplementary Table S1, shown as compound number as well as its alternative name).

The compound synthesis is shown in Figure 1C to 1E. The target compound **6** started from the commercially available 5-bromo-2-aminopyridine which was treated with 4-methoxybenzenesulfonyl chloride to produce compound **19**. The resulting product was reacted with *tert*-butyl acrylate under Heck reaction conditions and then the ester group was hydrolyzed to afford the corresponding acid **21**. The treatment of compound **21** with NH_2_OTHP, EDC‧HCl, and HOBt yielded the protected N-hydroxyacrylamide which was subsequently hydrolyzed by 10% TFA_(aq.)_ to afford the designed compound **6** (Figure 1C).

The general method for the synthesis of N-1 sulfonamides 6,5 membered ring with N-hydroxyacrylamides (1-arylsulfonyl-5-(*N*-hydroxyacrylamide)azaindolines) **7**-**13** are shown in Figure 1D. The preparation of compounds **7**-**13** started with a commercially available 7-azaindoline and underwent a four-step cascade sequence. The treatment of 7-azaindoline (**22**) with bromine yielded the corresponding bromo product **23**, which was reacted with various arylsulfonyl chlorides to afford compounds **24a** to **24g**. The following Heck olefination of **24a**-**24g** with methyl or *tert*-butyl acrylates has followed by ester hydrolysis, yielding compounds **25a** to **25g**. The resulting carboxylic acids underwent amide formation with NH_2_OTHP and deprotection with TFA to afford N-hydroxyacrylamides **7**-**13**.

The general method for the synthesis of N-1 sulfonamides 6,5 membered ring with N-hydroxyacrylamides (1-arylsulfonyl-5-(*N*-hydroxyacrylamide)azaindoles) **14**-**18** are shown in Figure 1E. The preparation of compounds **14**-**18** started with a commercially available 5-bromo-7-azaindole (**26**) which was treated with various commercially available substituted benzenesulfonyl chloride yielded the related 1-arylbenzenesulfonylazaindoles (**27a-27e**). These azaindoles-1-sulfonamides were subject to the heck reaction with *tert*-butyl acrylate followed by acid catalyzed hydrolysis to get compound **28a**-**28e**. Take these compounds undergoing the EDC‧HCl and HOBt-mediated amide coupling reaction, and the reaction was carried on TFA-mediated deprotection to afford the desired N-1 sulfonamides 6,5 membered ring with N-hydroxyacrylamides **14**-**18**.

According to our screening results of *TIMP3* inducers, compound **11** with its alternative name MPT0B390, exhibited the greatest increase in *TIMP3* mRNA level (Figure 1F) and induced apoptosis (Figure 1G) in HCT116 cells. In addition, MPT0B390 showed the most potent ability to inhibit HCT116 cell proliferation and broad-spectrum of HDAC inhibitory capabilities (Table 1 and Supplementary Table S2). Therefore, MPT0B390 is a potential *TIMP3* inducer and was chosen for further mechanism investigation.

### MPT0B390 inhibits colon cancer tumor growth *in vitro* and *in vivo*

**Figure 3:**
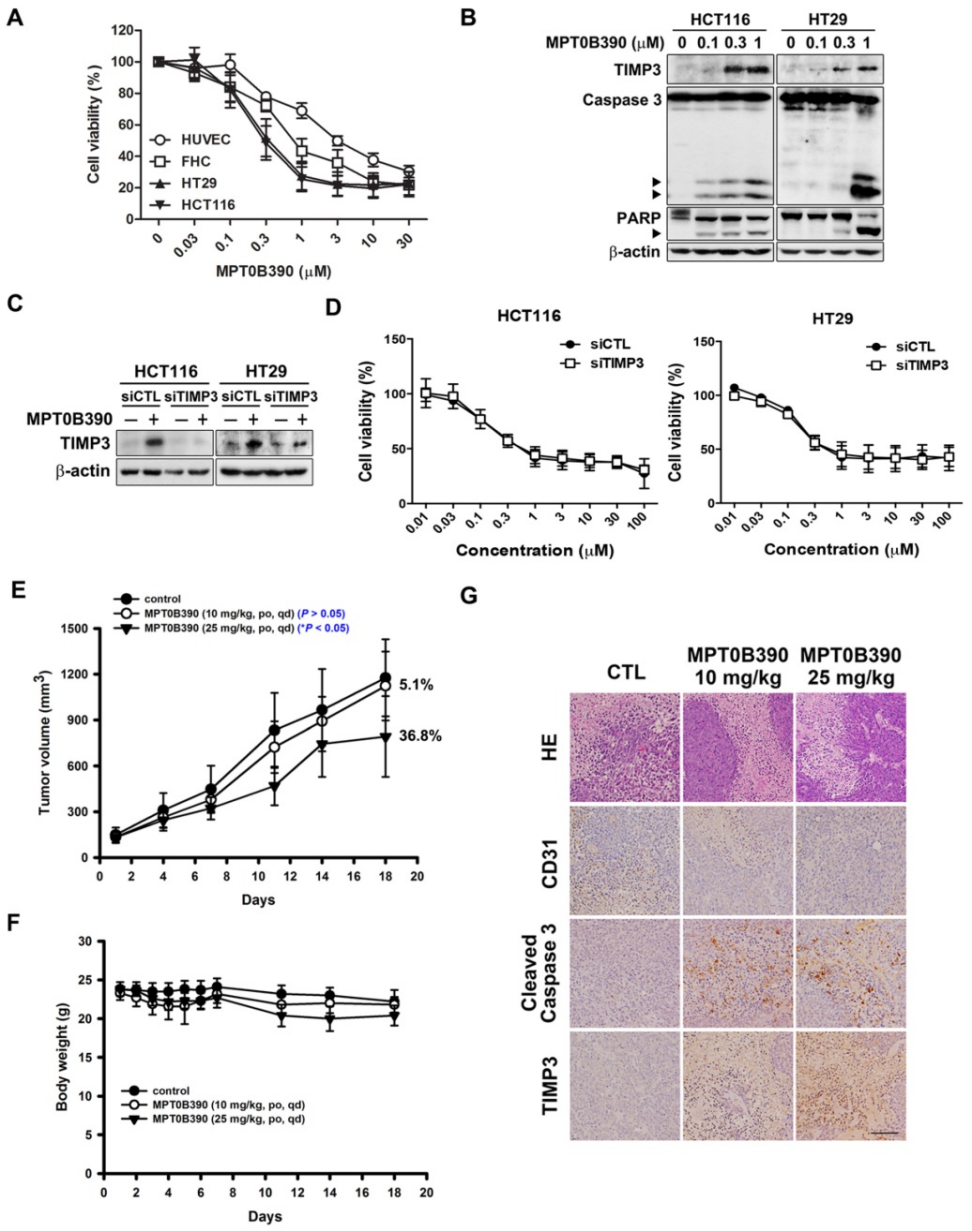
** MPT0B390 activates TIMP3 expression and apoptosis *in vitro* and *in vivo* in colon cancer.** (A) Cell viability of normal cell lines and colon cancer cell lines under MPT0B390 treatment. Cells were treated with indicated concentration of MPT0B390 for 48 h and viability was measured by MTT assay. Data are expressed as mean ± SEM of at least three independent experiments. (B) MPT0B390 induced apoptosis *in vitro*. Cells were treated with indicated concentration of MPT0B390 for 48 h and lysate were subjected to western blot. (C) TIMP3 knockdown efficiency in CRC cells. Cells were transfected with TIMP3 siRNA for 24 h followed by vehicle or MPT0B390 0.3 μM treatment for additional 48 h and subjected to western blot. (D) Cell viability assay of MPT0B390 in TIMP3 knockdown CRC cells. Cells were transfected with TIMP3 siRNA for 24 h followed by MPT0B390 treatment for another 48 h. Cell viability was measured using MTT assay. (E-G) Effect of MPT0B390 on the growth of HCT116 tumor xenografts in BALB/c nude mice. (E) Tumor growth volume curves were expressed as means ± S.D., and the percentage of TGI was determined. (*P < 0.05, compared with the control group) (F) Body weights were measured and expressed as means ± S.D. (G) Immunohistochemistry staining of xenograft tumors. Scales was shown as 50 μm. HE means hematoxylin and eosin staining.

First, we evaluated the cytotoxicity of MPT0B390 in normal epithelial cells (HUVEC and FHC) and CRC cells (HCT116 and HT29) using MTT assay. MPT0B390 showed concentration-dependent growth inhibition effect on CRC cells with IC_50_ values of 0.36±0.12 μM in HCT116 and 0.45±0.17 μM in HT29, but showed 2.5 to 9.6-fold higher IC_50_ values with HUVEC and FHC, respectively. This suggests its safety against normal cells (HUVEC, 3.48±1.00 μM; FHC, 1.15±0.44 μM) (Figure 3A). Also, MPT0B390 induced caspase 3 and PARP activation as well as TIMP3 expression in a concentration-dependent manner. Therefore, MPT0B390 is capable of inducing apoptosis in colorectal cancer cells (Figure 3B). In order to determine whether MPT0B390 inhibited cancer cell growth through *TIMP3* induction, we transiently transfected siRNA to decrease *TIMP3* endogenous gene expression (Figure 3C) and the capability of survival rescue under MPT0B390 treatment was performed. Knockdown of *TIMP3* expression could not reverse MPT0B390-inhibited CRC growth, indicating that MPT0B390 induced CRC apoptosis independent of *TIMP3* induction (Figure 3D). Next, we evaluated the antitumor effects of MPT0B390 in a HCT116 xenograft model. Our results showed that 25 mg/kg MPT0B390 could inhibit tumor growth (36.8% TGI) without influencing body weight (Figure 3E and F). Tumors were excised for hematoxylin and eosin (H&E) staining and immunohistochemical staining for the angiogenic marker CD31, apoptosis marker cleaved caspase 3, and TIMP3 expression. Administration of MPT0B390 decreased CD31-expression vessels, increased cleaved caspase 3 and TIMP3 expression significantly and dose-dependently (Figure 3G). These data indicated that MPT0B390 inhibit CRC cell growth partly by up-regulating TIMP3 *in vitro* and inhibit CRC growth *in vivo*.

### MPT0B390 inhibits tumor metastasis through TIMP3 activation

**Figure 4:**
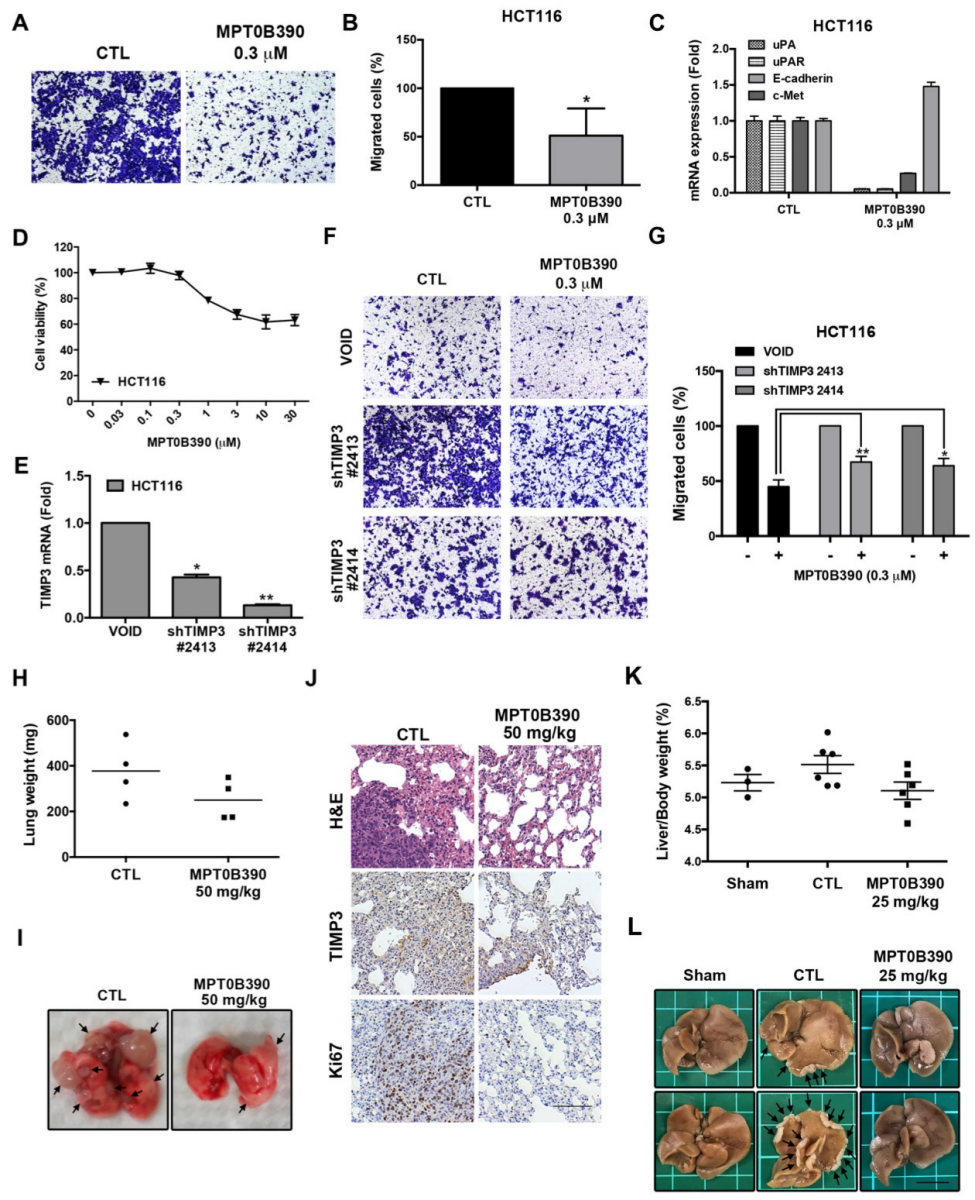
** MPT0B390 inhibits tumor metastasis through TIMP3 activation *in vitro* and *in vivo*.** (A, B) MPT0B390 inhibited CRC cell migration *in vitro*. HCT116 cells were seeded in transwell and treated with MPT0B390 0.3 μM for 24 h. (A) Migrated cells were stained and photographed. 100× magnification was used to observe via microscope. (B) Quantitative analysis of migrated cells. (C) real-time PCR analysis of migration-related genes in MPT0B390-treated HCT116 cells. (D) Cell viability assay of MPT0B390 in HCT116 after 24 h treatment. (E) Knockdown efficiency of shTIMP3 stable clones. HCT116 cells were infected with shTIMP3 lentivirus particles, selected and collected for mRNA detection using real-time PCR. (F, G) shTIMP3 stable clones were seeded in transwell and treated with MPT0B390 0.3 μM for 24 h. (F) Migrated shTIMP3 stable clone cells were stained and photographed. 100× magnification was used to observe via microscope. (G) Quantitative analysis of migrated shTIMP3 stable clone cells. (H-J) *In vivo* anti-metastasis effect of MPT0B390 in CT-26 inoculating animal model. Vehicle or MPT0B390 was given by oral gavage once daily during 3-week treatment. (H) Quantification of gross weight of lung tissues in each group (n=4). Black bar represents the average value. (I) Representative images of metastatic lung nodules from each group. Arrows indicate surface lung nodules. (J) Immunohistochemistry staining of lung slices in each group. Scale bar represents 100 μm. (K, L) *In vivo* anti-metastasis effect of MPT0B390 in HCT-116 inoculating liver metastasis animal model. Vehicle or MPT0B390 was given by oral gavage once daily during 3-week treatment. (K) Quantification of the percentage of liver gross weight over body weight in each group. Data is shown as mean ± S.E.M. (L) Representative images of metastatic liver nodules from each group. Arrows indicate surface liver nodules. Scale bar represents 1 cm.

Since TIMP3 is involved with metastasis 12, 23, we further examined the anti-metastasis effect of MPT0B390. First, we used a transwell migration assay to determine the effect of MPT0B390 on cancer cell motility *in vitro*. As shown in Figure 4A and 4B, 0.3 μM of MPT0B390 significantly inhibited HCT116 cell migration, while simultaneously downregulating gene expressions that regulate epithelial-mesenchymal transition (EMT) (e.g. *c-Met*, *uPA* and *uPAR*) and induce epithelial marker (e.g. *E-cadherin*) (Figure 4C).

Furthermore, 0.3 μM of MPT0B390 did not affect cell viability significantly while exerting its anti-metastatic effect in HCT116 (Figure 4D). Next, we used lentiviral transduction system to establish two *TIMP3* knockdown stable clones of HCT116 to further examine the role of TIMP3 in MPT0B390-inhibited cancer cell migration. *TIMP3* knockdown efficiency was confirmed by using real-time PCR (Figure 4E). The inhibitory effect of MPT0B390 on cancer cell motility was slightly rescued in the *TIMP3* knockdown stable clones, which indicated that MPT0B390 inhibited cancer cell migration through *TIMP3* induction *in vitro* (Figure 4F and G). Further, we generated lung tumor metastasis model and liver metastasis model to investigate the effect of MPT0B390 on cancer cell metastasis *in vivo*. Although no significant differences between lung weight or percentage of liver weight over body weight were observed between the control and the treated group, administration of MPT0B390 inhibited colorectal cancer cells migration to lung tissues as well as liver tissues. MPT0B390 did reduced the gross weight of lung tumors (Figure 4H), the percentage of liver gross weight over body weight (Figure 4K), and the numbers of tumor nodules (Figure 4I and 4L). Moreover, the anti-metastatic effect of MPT0B390 was corroborated in H&E staining of lung tissues. The tumor regions in pulmonary alveoli were significantly diminished with inhibition of proliferation marker Ki-67. In addition, TIMP3 expression was elevated in certain tumor areas in lung tissues treated with MPT0B390 (Figure 4J). Taken together, these results indicated that MPT0B390 suppresses tumor metastasis by up-regulating *TIMP3* expression *in vitro* and *in vivo*. These data also suggest that TIMP3 regulated tumor metastasis more than regulated tumor growth under MPT0B390 treatment.

### MPT0B390 transcriptionally induces TIMP3 expression by inhibiting EZH2 through reducing the binding of EZH2 to TIMP3 promoter region

**Figure 5:**
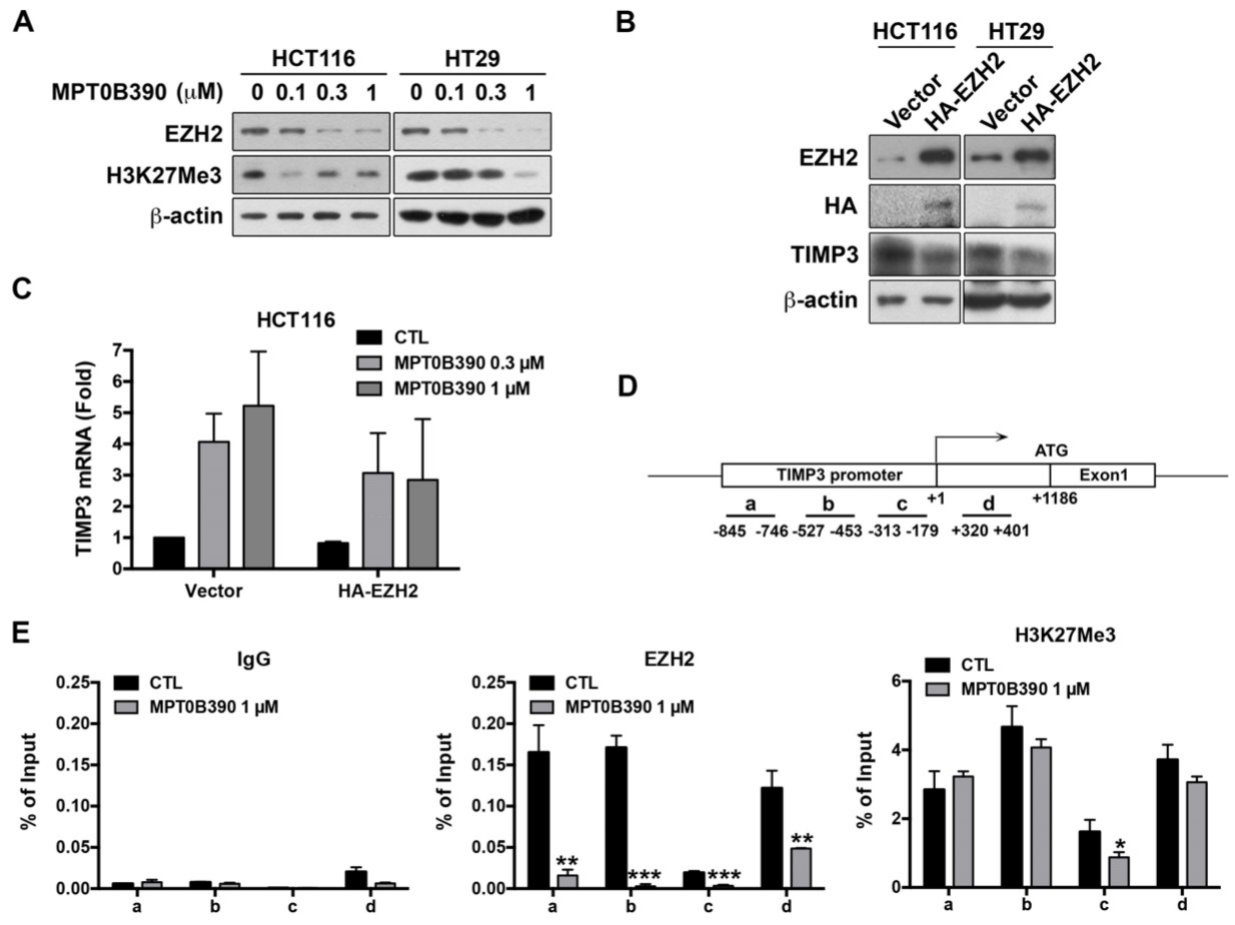
** EZH2 contributes to MPT0B390-induced TIMP3 activation.** (A) MPT0B390 inhibited EZH2 and H3K27Me3 concentration-dependently in CRC cells. Cells were treated with indicated concentration of MPT0B390 for 48 h and subjected to western blot analysis. (B) TIMP3 expression is negatively associated with EZH2 overexpression in CRC cells. Cells were transfected with vector or HA-EZH2 plasmid for 48 h and subjected to western blot analysis. (C) MPT0B390 induces TIMP3 expression partly through EZH2 downregulation. Cells were transfected with vector or HA-EZH2 plasmid for 24 h followed by MPT0B390 treatment for additional 24 h. *TIMP3* mRNA induction level was determined using real-time PCR. (D) Schematic representation of the promoter region of the *TIMP3* gene. The lines below the *TIMP3* locus represent the regions that amplified by real-time PCR with specific primer set (also see Supplementary Table S3). (E) MPT0B390 inhibits the binding of EZH2 on the *TIMP3* promoter region. HCT116 cells were treated with MPT0B390 for 24 h and then subjected to ChIP assay. Immunoprecipitated DNA was analyzed by real-time PCR analysis.

Since TIMP3 plays a major role in regulating tumor metastasis, we elucidate how MPT0B390 transcriptionally induces *TIMP3* expression. According to the literature, TIMP3 has been found to be epigenetically regulated by polycomb group protein enhancer of zeste homolog 2 (EZH2) through catalyzing the H3K27 trimethylation in the *TIMP3* promoter region and then subsequently decreases *TIMP3* transcription 24-26. As shown in Figure 5A, we found that MPT0B390 inhibited EZH2 expression as well as H3K27 trimethylation concentration-dependently in CRC cells. We transfected HA-tagged EZH2 plasmid to examine the role of EZH2 in MPT0B390-induced *TIMP3* expression. While ectopically transducing EZH2, TIMP3 protein expression was downregulated (Figure 5B), and MPT0B390-induced *TIMP3* mRNA expression was partially reversed upon EZH2 overexpression (Figure 5C). The *TIMP3* induction effect of MPT0B390 was further potentiated in EZH2 knockdown HCT116 cell (Supplementary Figure S2). These data indicated that EZH2 might be involved in regulating *TIMP3* transcription. Next, we performed ChIP assay in HCT-116 cells to examine the underlying mechanism of the transcriptional repression of *TIMP3* by EZH2. Since EZH2 is a component of the Polycomb Repressive Complex 2 (PRC2), and YY1 is the DNA binding protein that recruits PRC2 to DNA^24^, ^27^, we analyzed the immunoprecipitated DNA by real-time PCR with a designed primer set to amplify the regions containing the YY1 binding site in the *TIMP3* promoter (Figure 5C and Supplementary Table S3). We found that MPT0B390 strongly inhibited the binding of EZH2 to the *TIMP3* promoter region and slightly influenced the H3K27 trimethylation in the promoter region. Taken together, these data indicated that MPT0B390 induced *TIMP3* expression by inhibiting the expression of EZH2 as well as the binding of EZH2 to the *TIMP3* promoter region.

### MPT0B390 inhibits endothelial cell migration and angiogenesis *in vivo*

**Figure 6:**
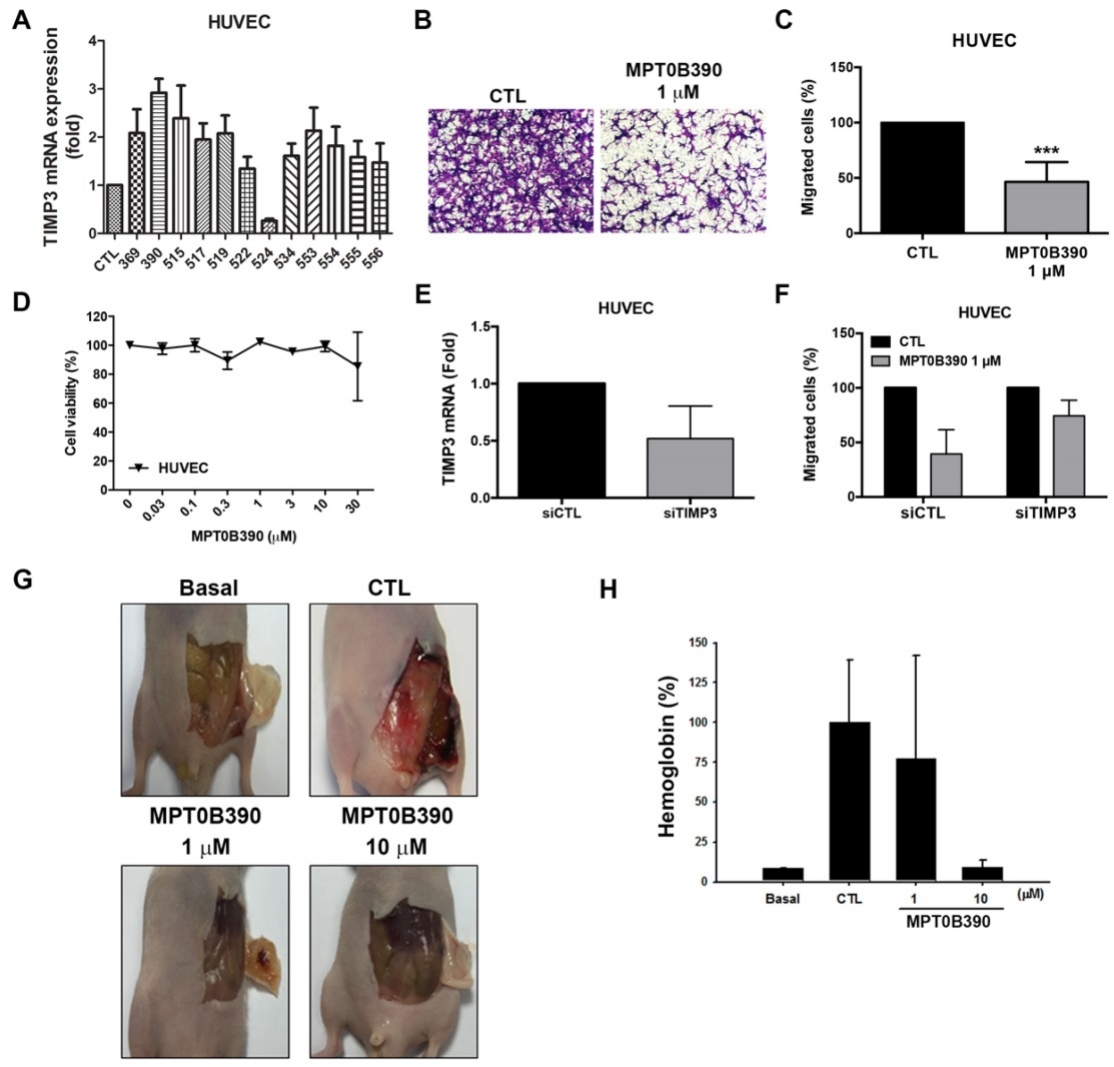
** MPT0B390 inhibits angiogenesis *in vitro* and *in vivo*.** (A) *TIMP3* mRNA levels activated by sulfonamide compounds. HUVEC cells were treated with 10 μM of indicated compounds for 24 h and mRNA was quantified by real-time PCR. (B, C) MPT0B390 inhibits migration of endothelium cells. HUVEC cells were treated with MPT0B390 for 24 h and then allowed to migrate for 6 h. (B) Migrated HUVEC cells were stained and photographed. 100× magnification was used to observe via microscope. (C) Quantification analysis of migrated HUVEC cells. (D) Cell viability of MPT0B390 after 24 h treatment. (E) TIMP3 knockdown efficiency in HUVEC cells. Cells were transfected with TIMP3 siRNA for 48 h and mRNA levels were determined using real-time PCR. (F) MPT0B390 inhibits endothelium cell migration partly through TIMP3 activation. HUVEC cells were transfected with TIMP3 siRNA for 24 h followed by MPT0B390 treatment for additional 24 h. Cells were then seeded in tranwell allowing for migration for 6 h. (G, H) MPT0B390 inhibits angiogenesis in Matrigel Plug assay. (G) Nude mice were injected subcutaneously with matrigel mixed with indicated condition. Plugs were excised from mice after a week and photographed. (H) Hemoglobin content were quantified by spectrophotometer measured at 540 nm. All data represent the mean ± SEM from at least independent experiments.

TIMP3 has been characterized as inhibitors of MMPs as an antiangiogenic protein 28, 29. Based on the *TIMP3* induction capability of MPT0B390, we next investigated the effect of MPT0B390 on tumor angiogenesis. MPT0B390 induced *TIMP3* mRNA expression most significantly among those *TIMP3* inducers in endothelial cells (Figure 6A). During angiogenesis, endothelium cell migration is triggered by chemotaxis, haptotaxis and mechanotaxis 30. We found that MPT0B390 inhibited endothelial cell motility without influencing cell viability (Figure 6B-D). In order to confirm that whether *TIMP3* is an important mediator of MPT0B390-inhibited angiogenesis, we knocked down *TIMP3* using siRNA and confirmed the knockdown efficiency by real-time PCR (Figure 6E). Knockdown *TIMP3* partially rescued MPT0B390-inhibited endothelial cell migration (Figure 6F). This suggests that MPT0B390 inhibits endothelial cell motility through *TIMP3* induction *in vitro*. Next, the anti-angiogenic effect of MPT0B390 was examined *in vivo* using matrigel plug assay. MPT0B390 significantly decreased vasculature in MPT0B390-infused gels compared to the growth-factor-only CTL plugs (Figure 6G). Quantification of hemoglobin contents further reveal that MPT0B390 inhibited angiogenic response in a concentration-dependent manner (Figure 6H). Taken together, these data suggest that MPT0B390 attenuated angiogenesis *in vitro* and *in vivo*, which is partly through *TIMP3* induction.

## Discussion

In this study, we demonstrated that MPT0B390 induces *TIMP3* to inhibit CRC tumor migration, invasion and angiogenesis *in vitro* and *in vivo*. Previous studies have shown that TIMP3 promotes apoptosis through stabilization of TNF- α receptors on the CRC cell surface, leading to increased susceptibility to apoptosis 7. TIMP3 expression also induces apoptosis initiators, such as capsase-8 and -9, to promote PARP cleavage through the Fas-associated death receptor-dependent apoptotic pathway 8. Although MPT0B390 strongly induced *TIMP3* mRNA and protein expression, as well as apoptosis activation in HCT116 cells (Figure 1F-G and Figure 3B), our findings show that it did not inhibit tumor cell survival through *TIMP3* induction according to the same viability in siRNA-mediated blockage of *TIMP3* upregulation (Figure 3C-D). This suggest that another mechanism is involved when CRC tumor growth is inhibited with MPT0B390. Indeed, MPT0B390 is an arylsulfonamide-based derivative with potent HDAC inhibitory ability (Supplementary Table S1 and S2). This may potentially play a role in MPT0B390-inhibited tumor growth by indirectly inhibiting tumor survival pathways. On the other hand, MPT0B390 can significantly inhibit tumor cell migration through *TIMP3*induction. We generated lentivirus-infected *TIMP3* knockdown HCT116 stable clones and found that MPT0B390-inhibited tumor cell migration can be abrogated by TIMP3 blockage (Figure 4E-G). TIMP-3 is unique among the four mammalian TIMPs and has the capability of inhibiting a broad spectrum of MMP and metalloproteinases, such as ADAM and ADAMTS families 29. Due to these characteristics of TIMP3, it was not surprising that MPT0B390, a *TIMP3* inducer, exerts anti-metastatic effects by inducing *TIMP3* expression. This also suggests that TIMP3 plays an important role in tumor metastasis rather than colorectal cancer survival. Previous research has identified TIMP3 as a tumor suppressor and can effectively inhibit tumor growth, metastasis, invasion and angiogenesis, which is partly due to TIMP3 inhibitory capacity to MMPs 22, 28, 31. However, TIMP3 has also been found to regulate cell fate and transformation by directing the balance between apoptosis and senescence in liver tumorigenesis 32. Therefore, detailed mechanisms of TIMP3 regulating early tumor development should be further elucidated. Nevertheless, according to our results, MPT0B390 can exert its anti-tumor effect in apoptosis induction, anti-metastasis and anti-angiogenesis, which displays the board therapeutic potential of MPT0B390.

TIMP3 expression has been reported to associate with malignant behaviors in various cancer types 13, 33 and can predict survival in hepatocellular carcinoma and breast cancer 33, 34. We examined TIMP3 expression in CRC patients and found that reduced TIMP3 expression is negatively associated with CRC prognosis. Based on these aspects, some studies used recombinant TIMPs or gene transfer systems to demonstrate that inhibiting metalloproteinases with TIMPs block tumor growth and invasion 35. However, some evidence has also shown that epigenetic silencing of *TIMP3* occurs in a variety of solid tumors 36-38, which indicates that upregulating TIMP3 expression directly may only have limited effect on its antitumor capability. MPT0B390, with its HDAC inhibitory activity, can epigenetically induce *TIMP3* expression by inhibiting EZH2 expression as well as the binding of EZH2 to *TIMP3* promoter. These data indicated that MPT0B390, a *TIMP3* inducer with epigenetic modulation ability, is a rational strategy against these kinds of solid tumors.

In conclusion, MPT0B390 exhibits tumor growth inhibition, as well as anti-metastasis and anti-angiogenesis activities. These functions indicate that MPT0B390 has potential use as a therapeutic agent for colorectal cancer treatment.

## Figures and Tables

**Table 1:** Antiproliferative activity against human colon cancer cell line HCT116 by compounds **7**-**18**

Compd	Alternative name	HCT116
GI_50_ (μM±SD)		
**14**	MPT0B369	0.06 ± 0.00
**7**	MPT0B390	0.03 ± 0.01
**9**	MPT0B515	0.09 ± 0.03
**8**	MPT0B517	0.17 ± 0.07
**10**	MPT0B519	0.14 ± 0.05
**11**	MPT0B522	5.1 ± 0.7
**12**	MPT0B524	5.46 ± 0.9
**13**	MPT0B534	2.91 ± 1.01
**15**	MPT0B553	0.07 ± 0.03
**16**	MPT0B554	1.58 ± 1.05
**17**	MPT0B555	1.65 ± 0.31
**18**	MPT0B556	5.46 ± 0.81

*^a^*SD: standard deviation. All experiments were independently performed at least three times.

**Table 2:** Association of TIMP3 expression and clinical parameters in tumor tissues of colorectal cancer patients.

	TIMP3		
Parameters	Low (n=113) (%)	High (n=79) (%)	p value
Age (years)			
≦65	54 (47.8)	40 (50.6)	
>65	59 (52.2)	39 (49.4)	0.770
Gender			
Female	47 (41.6)	43 (54.4)	
Male	66 (58.4)	36 (45.6)	0.106
T factor			
1	4 (3.5)	3 (3.8)	
2	11 (9.7)	17 (21.5)	
3	67 (59.3)	43 (54.4)	
4	31 (27.4)	16 (20.3)	0.136
N factor			
0	42 (37.2)	41 (51.9)	
1+2	71 (62.8)	38 (48.1)	0.054
M factor			
0	88 (77.9)	71 (89.9)	
1	25 (22.1)	8 (10.1)	0.003
Stage			
I	6 (5.3)	17 (21.5)	
II	29 (25.7)	24 (30.4)	
III	52 (46.0)	30 (40.0)	
IV	26 (23.0)	8 (10.1)	0.001

No positive or only one positively stained section was defined as “low” expression, and the presence of at least two positively stained sections was classified as “high” expression (N = 192).

## Abbreviations

TIMP3: Tissue inhibitors of metalloproteinase 3
MMP: matrix metalloproteinase
CRC: colorectal cancer
ECM: extracellular matrix
ADAM: a disintegrin and metalloproteinase
VEGF: vascular endothelial growth factor
HDAC: histone deacetylase
SRB: Sulforhodamine B
MTT: 3-(4,5-Dimethylthiazol-2-yl)-2,5-diphenyltetrazolium bromide
EMT: epithelial-mesenchymal transition
