# TIMP3 expression associates with prognosis in colorectal cancer and its novel arylsulfonamide inducer, MPT0B390, inhibits tumor growth, metastasis and angiogenesis: Erratum

**DOI:** 10.7150/thno.56734

**Published:** 2021-01-01

**Authors:** Han-Li Huang, Yi-Min Liu, Ting-Yi Sung, Tsui-Chin Huang, Ya-Wen Cheng, Jing-Ping Liou, Shiow-Lin Pan

**Affiliations:** 1 TMU Biomedical Commercialization Center, Taipei Medical University, Taipei 11031, Taiwan; 2 Ph.D Program in Biotechnology Research and Development, College of Pharmacy, Taipei Medical University, Taipei 11031, Taiwan.; 3 Graduate Institute of Cancer Molecular Biology and Drug Discovery, College of Medical Science and Technology, Taipei Medical University, Taipei 11031, Taiwan.; 4 School of Pharmacy, College of Pharmacy, Taipei Medical University, Taipei 11031, Taiwan.

The authors wish to inform readers of a correction to the study. The informed consent and the IRB approval number in the Materials and methods section, should be revised as follows:

The human tissue biopsy samples used in this study were collected as part of a study approved by the human ethics committee of Taipei Medical University Joint Institutional Review Board (TMU-JIRB No. N201701061). The waiver of informed consent for the study was monitored by TMU-JIRB.

The authors regret the inconsistency and apologize for not identifying the issue earlier in the publication process.

