# Supplementary material for: TIMP3 expression associates with prognosis in colorectal cancer and its novel arylsulfonamide inducer, MPT0B390, inhibits tumor growth, metastasis and angiogenesis
Source: Theranostics. 2019 Sep 18;9(22):6676–89. doi: 10.7150/thno.34020 (PMC6771239; doi:10.7150/thno.34020)
Supplement: Supplementary file 1 — Supplementary figures and tables. [file thnov09p6676s1.pdf]

TIMP3 expression associates with prognosis in colorectal cancer and its novel arylsulfonamide inducer, MPT0B390, inhibits tumor growth, metastasis and angiogenesis

Han-Li Huang[1,2], Yi-Min Liu[1], Ting-Yi Sung[2,] Tsui-Chin Huang[3], Ya-Wen Cheng[3], Jing-Ping Liou[1, 4*], Shiow-Lin Pan[1,2,3,*]

[1] *TMU Biomedical Commercialization Center, Taipei Medical University, Taipei 11031, Taiwan*

[2] *Ph.D Program in Biotechnology Research and Development, College of Pharmacy, Taipei Medical University, Taipei 11031, Taiwan.*

[3] *Graduate Institute of Cancer Molecular Biology and Drug Discovery, College of Medical Science and Technology, Taipei Medical University, Taipei 11031, Taiwan.*

[4] *School of Pharmacy, College of Pharmacy, Taipei Medical University, Taipei 11031, Taiwan.*

***Correspondence:**

Shiow-Lin Pan, Ph.D.
The Ph.D. Program for Cancer Biology and Drug Discovery,
College of Medical Science and Technology, Taipei Medical University,
No. 250 Wuxing Street, Taipei 11031, Taiwan
E-mail: slpan@tmu.edu.tw
Phone: 886-2-27361661 ext 7671

Jing-Ping Liou, Ph.D.
School of Pharmacy, College of Pharmacy, Taipei Medical University, Taipei, Taiwan
No. 250, Wuxing St., Taipei 11031, Taiwan.
E-mail address: jpl@tmu.edu.tw
Phone: 886-2-27361661 ext 6130

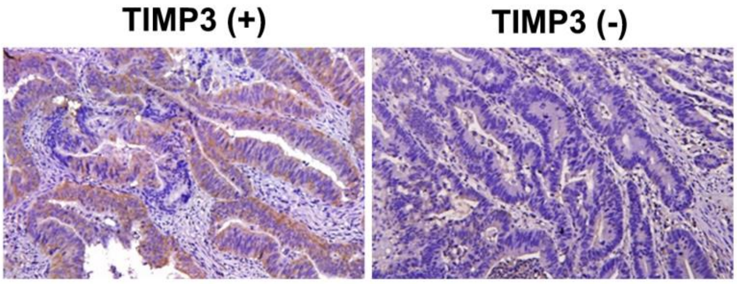

Supplementary Figure S1. Representative TIMP3 expression in colon cancer patients (Patients' information and Kaplan-Meier analysis please see Table 2, and Figure 2A and B, respectively).

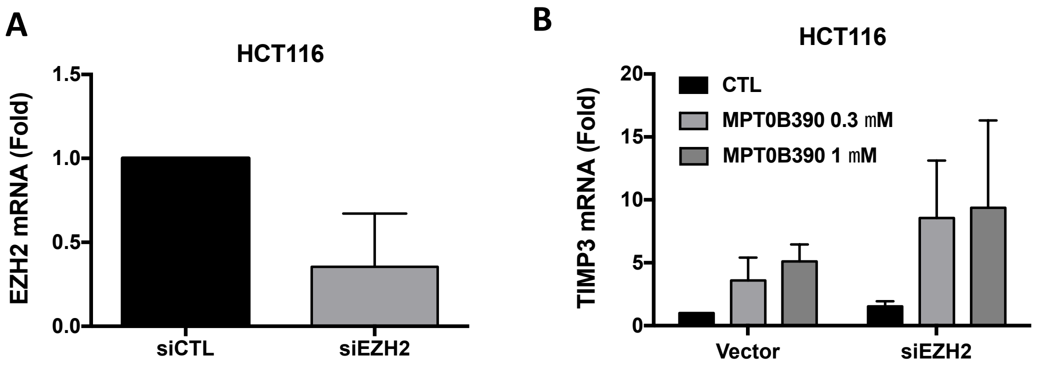

Supplementary Figure S2. Effect of MPT0B390 on *TIMP3* induction in EZH2 knockdown HCT116 cell. (A) Knockdown efficiency of siEZH2 in HCT116 cells. HCT116 cells were transfected with siEZH2 for 24 h and then collected for mRNA detection using real-time PCR. (B) MPT0B390 further induced *TIMP3* expression in EZH2 knockdown HCT116 cells. HCT116 cells were transfected with siEZH2 for 24 h and treated with MPT0B390 for an additional 24h. Cells were then collected for mRNA detection using real-time PCR.

Supplementary Table S1. Inhibition of HeLa nuclear extract HDAC activity by compounds **7-18**

Compd	Alternative name	HeLa nuclear HDACs
	IC_{50} (μM)	
**14**	MPT0B369	0.078
**7**	MPT0B390	0.004
**9**	MPT0B515	0.050
**8**	MPT0B517	0.060
**10**	MPT0B519	0.030
**11**	MPT0B522	0.008
**12**	MPT0B524	0.003
**13**	MPT0B534	0.170
**15**	MPT0B553	0.032
**16**	MPT0B554	0.007
**17**	MPT0B555	0.008
**18**	MPT0B556	0.1

Supplementary Table S2. Activities of MPT0B390 and reference Trichostatin A against HDAC isoforms 1, 2, 3, 4, 5, 6, 7, 8, 9 and 11

	IC_{50} (nM)	
Isoenzyme	MPT0B390	Trichostatin A
HDAC1	17.2	14.5
HDAC2	10.1	11.8
HDAC3	4.16	17.9
HDAC4	-	5870.0
HDAC5	-	1440.0
HDAC6	20.9	3.96
HDAC7	-	1490.0
HDAC8	72.2	148.0
HDAC9	-	8860.0
HDAC11	19.9	30.7

Supplementary Table S3. Primer sequences for qRT-PCR and chromatin immunoprecipitation assays.

**A. qRT-PCR**	
TIMP3	5'-TGCTCTCTGTCTCTTTTTTCAGCTT-3' (Foward)<br>5'-CTACAGTGTGTTGTCTGCTGCTTTT-3' (Reverse)
uPA	5'-CCACAACGACATTGCCTTGCTGAA-3' (Foward)<br>5'-ATCGTTATACATCGAGGGCAGGCA-3' (Reverse)
uPAR	5'-AAGATCACCAGCCTTACCGAGGTT-3' (Foward)<br>5'-ATTCGAGGTAACGGCTTCGGGAAT-3' (Reverse)
c-Met	5'-ATCAACATGGCTCTAGTTGTC-3' (Foward)<br>5'-GGGAGAATATGCAGTGAACC-3' (Reverse)
E-cadherin	5'-ATTCTGATTCTGCTGCTCTTG-3' (Foward)<br>5'-AGTCCTGGTCCTCTTCTCC-3' (Reverse)
GAPDH	5'-TGGGATTTCCATTGATGACAAG-3' (Foward)<br>5'-ATTCCACCCATGGCAAATTC-3' (Reverse)
18S rRNA	5'-AACCCGTTGAACCCCATT-3' (Foward)<br>5'-CCATCCAATCGGTAGTAGC-3' (Reverse)
**B. qRT-PCR primers for ChIP assay**	
TIMP3<br>Set a	5'-GTTAGAGTGAAGGCAGGGAAG-3' (Foward)<br>5'-TTTGAGCACTTTAGCCATAGGA-3' (Reverse)
TIMP3<br>Set b	5'-TGGAAGATCCCAGCTGCAAAT-3' (Foward)<br>5'-GAGACATGCATTTCCCTGAACG-3' (Reverse)
TIMP3<br>Set c	5'-GTGAGAGCTATAATACGGTGAGATAC-3' (Foward)<br>5'-CAGGATCCTAGACGACTAGCTATAA-3' (Reverse)
TIMP3<br>Set d	5'-CAGCAGATGGCTTCCCATATC-3' (Foward)<br>5'-CACAGAGGAGAAAGACCCAAAC-3' (Reverse)

Supporting information

N-(5-Bromopyridin-2-yl)-4-methoxybenzenesulfonamide (**20**). A mixture of 2-amino-5-bromopyridine (**19**, 1.05 g, 6.07 mmol), DMAP (0.07 g, 0.55 mmol), ACN (14 mL) and pyridine (3.6 mL) was added 4-methoxybenzensulfonyl chloride (1.27 g, 6.13 mmol) under nitrogen and stirred at rt. Overnight. The reaction was quenched with water and extracted with ethyl acetate (30 mL x 3). The organic layer was collected and dried over anhydrous $MgSO_4$ and concentrated *in vacuo* to yield a dark brown product. The residue was purified by flash column over silica gel (ethyl acetate: n-hexane = 1:1) to afford **20** (1.08 g, 51.84%) as a yellow solid. ^{1}H-NMR (300MHz, $CDCl_3$): δ 3.86 (s, 3H), 6.93(d, *J* = 9.0 Hz, 2H), 7.48 (d, *J* = 9.0 Hz, 1H), 7.73-7.81 (m, 3H), 8.40 (s, 1H).

(*E*)-3-(6-(4-Methoxyphenylsulfonamido)7xpressi-3-yl)acrylic acid (**21**). A mixture of **20** (0.14 g, 0.41 mmol), palladium acetate (0.01 g, 0.04 mmol), triphenylphosphine (0.02 g, 0.08 mmol), triethylamine (0.06 mL, 0.41 mmol), sodium bicarbonate (0.07 g, 0.82 mmol) and DMF (1.5 mL) was stirred for a while then added the *t*-butyl acrylate (0.07 mL, 0.49 mmol) at 120 ^{o}C and stirred overnight. The residue was purified by flash column over silica gel (ethyl acetate: n-hexane = 1:2) to give the *t*-butyl acrylate compound as a yellow solid. To the ester compound was added trifluoroacetic acid (1.5 mL) and stirred at room temperature for 1 hour. The reaction

was quenched with water and filtered by gravity filtration to yield a as a white solid **21** without further purification (0.21g, 61.55%). ^{1}H-NMR (300 MHz, CD_3OD + DMSO-d_6): δ 3.90 (s, 3H), 6.51 (d, *J* = 15.9 Hz, 1H), 7.12 (d, *J* = 8.7 Hz, 2H), 7.26 (d, *J* = 9.0 Hz, 1H), 7.60 (d, *J* = 15.9 Hz, 1H), 7.96 (d, *J* = 8.7 Hz, 2H), 8.06 (d, *J* = 9.0 Hz, 1H), 8.35 (s, 1H).

(*E*)-N-Hydroxy-3-(6-(4-methoxyphenylsulfonamido)pyridin-3-yl)acrylamide (6). A mixture of **21** (0.30 g, 0.90 mmol), EDC•HCl (0.26 g, 1.35 mmol), HOBt (0.15 g, 1.08 mmol), NMM (0.24 mL, 2.16 mmol) and DMF (2 mL) was stirred for a while then added the NH_2OTHP (0.13 g, 1.08 mmol) at room temperature and was stirred overnight. The residue was purified by flash column over silica gel (ethyl acetate: n-hexane = 4:1) to afford the oily product. The resulting oily product was dissolved in MeOH (5 mL) and added the 10% $TFA_{(aq.)}$ (5 mL) at room temperature and stirred for overnight. The reaction was added H_2O to produce the precipitant. The residue was without more purification to afford **6** (0.17g, 54.16%) as a white solid with m.p. 180.0-181.0 ^{o}C. ^{1}H-NMR (300MHz, DMSO-d_6): δ 3.79 (s, 3H), 6.33 (d, *J* = 15.9 Hz, 1H), 7.06 (d, *J*= 9.0 Hz, 2H), 7.12 (d, *J*= 9.0 Hz, 1H), 7.35 (d, *J*=15.9 Hz, 1H), 7.82 (t, *J*= 9.0 Hz, 2H), 7.89 (d, *J*= 9.0 Hz, 1H), 8.25 (s, 1H), 9.01 (s, 1H), 10.72 (s, 1H). ^{13}C-NMR (125MHz, DMSO-d_6): δ 55.61, 112.78, 114.17, 118.76, 128.98, 132.69,

134.22, 136.69, 152.70, 162.30, 162.52. HRMS (ESI) for $C_{15}H_{15}N_3O_5S$ ($[M+H]^+$): calcd, 349.0730; found, 350.0803.

5-bromo-2,3-dihydro-1H-pyrrolo[2,3-b]pyridine (**23**). To a mixture of 7-azaindoline (**22**, 4.0 g, 33.29 mmol), pyridine (4 mL) and CH_2Cl_2 (40 mL) was slowly added Br_2 (1.72 mL, 33.29 mmol) in CH_2Cl_2 (24 mL) under ice bath and was allowed to stir for 2 h under nitrogen. The reaction was quenched with water and extracted with ethyl acetate (30 mL x 3). The organic layer was collected and dried over anhydrous $MgSO_4$ and concentrated *in vacuo* to yield a dark brown product. The residue was purified by flash column over silica gel (ethyl acetate: n-hexane = 2:1) to afford **23** (4.88 g, 73.65%) as a yellow solid. ^{1}H-NMR (500 MHz, $CDCl_3$): δ 3.06 (t, *J* = 8.5 Hz, 2H), 3.64 (t, *J* = 8.5 Hz, 2H), 7.31 (s, 1H), 7.85 (s, 1H).

5-Bromo-1-(4-methoxyphenylsulfonyl)-2,3-dihydro-1H-pyrrolo[2,3-b]pyridine (24a). A mixture of **23** (0.35 g, 1.76mmol), 4-methoxybenzenesulfonyl chloride (0.55 g, 2.64 mmol) and pyridine (3 mL) was refluxed overnight. The reaction was quenched with water and extracted with ethyl acetate (30 mL x 3). The organic layer was collected and dried over anhydrous $MgSO_4$ and concentrated *in vacuo* to yield a brown product. The residue was purified by flash column over silica gel (ethyl acetate: n-hexane = 1:1) to afford **24a** (0.34 g, 52.31%) as a yellow solid. ^{1}H-NMR

(500 MHz, $CDCl_3$): δ 3.02 (t, *J* = 8.5 Hz, 2H), 3.84 (s, 3H), 4.05 (t, *J* = 8.5 Hz, 2H), 6.94 (d, *J* = 9.5 Hz, 2H), 7.45 (s, 1H), 8.01 (d, *J* = 8.0 Hz, 2H), 8.18 (s, 1H).

5-Bromo-1-(phenylsulfonyl)-2,3-dihydro-1H-pyrrolo[2,3-b]pyridine (24b). The title compound was obtained in 78.73% overall yield from compound **23** and benzenesulfonyl chloride in a similar manner as described for the preparation of **24b**. ^{1}H-NMR (500 MHz, $CDCl_3$): δ 3.04 (t, *J* = 8.5 Hz, 2H), 4.08 (t, *J* = 8.5 Hz, 2H), 7.46 (s, 1H), 7.49 (t, *J* = 8.0 Hz, 2H), 7.58 (t, *J* = 7.5 Hz, 1H), 8.08 (t, *J* = 8.0 Hz, 2H), 8.18 (s, 1H).

5-Bromo-1-(3-methoxyphenylsulfonyl)-2,3-dihydro-1H-pyrrolo[2,3-b]pyridine (24c). The title compound was obtained in 86.17% overall yield from compound **23** and 3-methoxybenzenesulfonyl chloride in a similar manner as described for the preparation of **24c**. ^{1}H-NMR (500 MHz, $CDCl_3$): δ 3.04 (t, *J* = 8.5 Hz, 2H), 3.84 (s, 3H), 4.07 (t, *J* = 8.5 Hz, 2H), 7.11 (dd, *J* = 2.5, 9.0 Hz, 1H), 7.38 (t, *J* = 8.0 Hz, 1H), 7.46 (s, 1H), 7.62 (dd, *J* = 1.5, 8.0 Hz, 1H), 7.65 (s, 1H), 8.19 (s, 1H).

5-Bromo-1-(3-nitrophenylsulfonyl)-2,3-dihydro-1H-pyrrolo[2,3-b]pyridine (24d). The title compound was obtained in 53.24% overall yield from compound **23** and 3-nitrobenzenesulfonyl chloride in a similar manner as described for the preparation of **24d**. ^{1}H-NMR (500 MHz, $CDCl_3$): δ 3.11 (t, *J* = 8.5 Hz, 2H), 4.16 (t, *J* = 8.5 Hz,

2H), 7.51 (s, 1H), 7.72 (t, *J* = 9.0 Hz, 1H), 8.18 (s, 1H), 8.42-8.44 (m, 1H), 8.46-8.49 (m, 1H), 8.96 (t, *J* = 9.0 Hz, 1H).

5-Bromo-1-(4-bromophenylsulfonyl)-2,3-dihydro-1H-pyrrolo[2,3-b]pyridine (24e). The title compound was obtained in 77.46% overall yield from compound **23** and 4-bromobenzenesulfonyl chloride in a similar manner as described for the preparation of **24e**. ^{1}H-NMR (500 MHz, $CDCl_3$): δ 3.05 (t, *J* = 8.5 Hz, 2H), 4.06 (t, *J* = 8.5 Hz, 2H), 7.47 (s, 1H), 7.62 (d, *J* = 8.5 Hz, 2H), 7.95 (d, *J* = 8.5 Hz, 2H), 8.16 (s, 1H).

5-Bromo-1-(3-bromophenylsulfonyl)-2,3-dihydro-1H-pyrrolo[2,3-b]pyridine (24f). The title compound was obtained in 76.10% overall yield from compound **23** and 3-bromobenzenesulfonyl chloride in a similar manner as described for the preparation of **24f**. ^{1}H-NMR (500 MHz, $CDCl_3$): δ 3.07 (t, *J* = 8.5 Hz, 2H), 4.09 (t, *J* = 8.5 Hz, 2H), 7.37 (t, *J* = 8.0 Hz, 1H), 7.48-7.49 (m, 1H), 7.69-7.71 (m, 1H), 8.03-8.05 (m, 1H), 8.19 (d, *J* = 9.0 Hz, 1H), 8.25 (d, *J* = 8.0 Hz, 1H).

5-Bromo-1-(2-bromophenylsulfonyl)-2,3-dihydro-1H-pyrrolo[2,3-b]pyridine (24g). The title compound was obtained in 40.77% overall yield from compound **23** and 2-bromobenzenesulfonyl chloride in a similar manner as described for the preparation of **24g**. ^{1}H-NMR (500 MHz, $CDCl_3$): δ 3.18 (t, *J* = 8.5 Hz, 2H), 4.49 (t, *J*

= 8.5 Hz, 2H), 7.38-7.42 (m, 1H), 7.48-7.52 (m, 2H), 7.67 (t, *J* = 8.0 Hz, 1H), 7.92 (s, 1H), 8.43 (d, *J* = 8.0 Hz, 1H).

(*E*)-3-(1-(4-Methoxyphenylsulfonyl)-2,3-dihydro-1H-pyrrolo[2,3-b]pyridin-5-yl) acrylic acid (24a). The title compound was obtained in 43.43% overall yield from compound **25a** in a similar manner as described for the preparation of **21.** ^{1}H-NMR (500 MHz, CD_3OD): δ 3.09 (t, *J* = 8.5 Hz, 2H), 3.83 (s, 3H), 4.07 (t, *J* = 8.5 Hz, 2H), 6.42 (d, *J* =16.0 Hz, 1H), 7.05 (d, *J* = 9.0 Hz, 2H),7.58 (d, *J* =16.0 Hz, 1H), 7.86 (s, 1H), 7.97 (d, *J* = 9.0 Hz, 2H), 8.16 (s, 1H).

(*E*)-3-(1-(Phenylsulfonyl)-2,3-dihydro-1H-pyrrolo[2,3-b]pyridin-5-yl)acrylic acid (25b). A mixture of **24b** (0.47 g, 1.39 mmol), palladium acetate (0.03 g, 0.14 mmol), triphenylphosphine (0.07 g, 0.28 mmol), triethylamine (0.19 mL, 1.39mmol), sodium bicarbonate (0.23 g, 2.78 mmol) and DMF (3 mL) was stirred for a while then added the methyl acrylate (0.15 mL, 1.67 mmol) at 120 ^{o}C and stirred for overnight. The residue was purified by flash column over silica gel (ethyl acetate: n-hexane = 1:1) to give the methyl acrylate compound as a yellow solid. A mixture of ester compound and dioxane (5 mL) was added 1M $LiOH_{(aq.)}$ (2.22 mL, 2.22 mmol) and stirred overnight at 40 ^{o}C. The reaction was removed out solvent and dissolved in water. The water layer was added the 3N $HCl_{(aq.)}$ and filtered by gravity filtration to yield a white product **25b** (0.36 g, 98.17%). ^{1}H-NMR (500 MHz, DMSO-d_6): δ 3.08 (t, *J* = 8.5 Hz,

2H), 4.08 (t, *J* = 8.5 Hz, 2H), 6.44 (d, *J* =16.0 Hz, 1H), 7.50 (d, *J* =16.0 Hz, 1H), 7.58 (t, *J* = 7.5 Hz, 2H), 7.68 (t, *J* = 7.5 Hz, 1H), 7.94 (s, 1H), 8.01 (d, *J* = 8.5 Hz, 2H), 8.26 (s, 1H).

(*E*)-3-(1-(3-Methoxyphenylsulfonyl)-2,3-dihydro-1H-pyrrolo[2,3-b]pyridin-5-yl) acrylic acid (25c). The title compound was obtained in 93.32% overall yield from compound **24c** in a similar manner as described for the preparation of **21**. ^{1}H-NMR (500 MHz, CD_3OD): δ 3.09 (t, *J* = 8.5 Hz, 2H), 3.82 (s, 3H), 4.08 (t, *J* = 8.5 Hz, 2H), 6.42 (d, *J* = 16.0 Hz, 1H), 7.18 (dd, *J* = 2.0, 8.5 Hz, 1H), 7.44 (t, *J* = 8.5 Hz, 2H), 7.57-7.61 (m, 3H), 7.83 (s, 1H), 8.19 (s, 1H).

(*E*)-3-(1-(3-Nitrophenylsulfonyl)-2,3-dihydro-1H-pyrrolo[2,3-b]pyridin-5-yl)-acrylic acid (25d). The title compound was obtained in 45.05% overall yield from compound **24d** in a similar manner as described for the preparation of **21**. ^{1}H-NMR (500 MHz, DMSO-d_6): δ 3.10 (t, *J* = 8.5 Hz, 2H), 4.15 (t, *J* = 8.5 Hz, 2H), 6.46 (d, *J* = 16.0 Hz, 1H), 7.51 (d, *J* = 16.0 Hz, 1H), 7.90 (t, *J* = 8.0 Hz, 1H), 7.97 (s, 1H), 8.29 (s, 1H), 8.44 (d, *J* = 8.0 Hz, 1H), 8.50 (d, *J* = 8.0 Hz, 1H), 8.74 (s, 1H).

(*E*)-4-(4-(5-((*E*)-2-Carboxyvinyl)-2,3-dihydro-1H-pyrrolo[2,3-b]13xpressi-1-ylsulfonyl)-phenyl)but-2-enoic acid (25e). The title compound was obtained in 42.33% overall yield from compound **24e** in a similar manner as described for the preparation of **21**. ^{1}H-NMR (500 MHz, DMSO-d_6): δ 3.09 (t, *J* = 8.5 Hz, 2H), 4.10 (t, *J* = 8.5 Hz,

2H), 6.45 (d, *J* = 16.0 Hz, 1H), 6.65 (d, *J* = 16.0 Hz, 1H), 7.50 (d, *J* = 16.0 Hz, 1H), 7.59 (d, *J* = 16.5 Hz, 1H), 7.88 (d, *J* = 8.5 Hz, 2H), 7.95 (s, 1H), 8.01 (d, *J* = 8.0 Hz, 2H), 8.27 (s, 1H).

(*E*)-3-(3-(5-((*E*)-2-Carboxyvinyl)-2,3-dihydro-1H-pyrrolo[2,3-b]14xpressi-1-ylsulfonyl)-phenyl)but-2-enoic acid (25f). The title compound was obtained in 35.35% overall yield from compound **24f** in a similar manner as described for the preparation of **21**. ^{1}H-NMR (500 MHz, DMSO-d_6): δ 3.09 (t, *J* = 8.5 Hz, 2H), 4.15 (t, *J* = 8.5 Hz, 2H), 6.44 (d, *J* = 16.0 Hz, 1H), 6.62 (d, *J* = 16.0 Hz, 1H), 7.49 (d, *J* = 16.0 Hz, 1H), 7.61-7.66 (m, 2H), 7.95 (s, 1H), 8.02 (t, *J* = 9.0 Hz, 2H), 8.21 (s, 1H), 8.26 (s, 1H).

(*E*)-3-(2-(5-((*E*)-2-Carboxyvinyl)-2,3-dihydro-1H-pyrrolo[2,3-b]14xpressi-1-ylsulfonyl)phenyl)acrylic acid (25g). The title compound was obtained in 33.42% overall yield from compound **24g** in a similar manner as described for the preparation of **25b**. ^{1}H-NMR (500 MHz, DMSO-d_6): δ 3.11 (t, *J* = 8.5 Hz, 2H), 4.32 (t, *J* = 8.5 Hz, 2H), 6.39-6.43 (m, 2H), 7.45 (d, *J* = 16.0 Hz, 1H), 7.64 (t, *J* = 8.0 Hz, 1H), 7.70 (t, *J* = 8.0 Hz, 1H), 7.86 (d, *J* = 7.5 Hz, 1H), 7.95 (s, 1H), 8.07 (s, 1H), 8.14 (d, *J* = 8.0 Hz, 1H), 8.33 (d, *J* = 16.0 Hz, 1H).

(*E*)-*N*-Hydroxy-3-(1-(4-methoxyphenylsulfonyl)-2,3-dihydro-1H-pyrrolo[2,3-b]pyridin-5-yl)acrylamide (7). The title compound was obtained in 88.79% overall yield from compound **25a** in a similar manner as described for the preparation of **6** with

m.p. 201.6-202.6 ^{o}C. ^{1}H-NMR (500 MHz, DMSO-d_6): δ 3.06 (t, *J* = 8.5 Hz, 2H), 3.80 (s, 3H), 4.01 (t, *J* = 8.5 Hz, 2H), 6.34 (d, *J* =16.0 Hz, 1H), 7.08 (d, *J* = 9.0 Hz, 2H), 7.38 (d, *J* =16.0 Hz, 1H), 7.72 (s, 1H), 7.93 (t, *J* = 9.0 Hz, 2H), 8.19 (s, 1H), 8.99 (br, 1H), 10.70 (s, 1H). ^{13}C-NMR (125MHz, DMSO-d_6): δ 24.44, 48.72, 55.68, 114.28, 118.48, 125.49, 125.54, 128.89, 129.97, 130.59, 134.88, 147.10, 156.32, 162.58, 163.21. MS (ESI) *m*/*z*: 376.0 ([M+H]$^{+}$). HRMS (ESI) for $C_{17}H_{17}N_3O_5S$ ([M-H]$^{-}$): calcd, 374.0816; found, 374.0823.

(*E*)-N-hydroxy-3-(1-(phenylsulfonyl)-2,3-dihydro-1H-pyrrolo[2,3-b]pyridin-5-yl) acrylamide (8). The title compound was obtained in 89.09% overall yield from compound **25b** in a similar manner as described for the preparation of **6** with m.p. 198.1-199.0 ^{o}C. ^{1}H-NMR (500MHz, DMSO-d_6): δ 3.08 (t, *J* = 8.5 Hz, 2H), 4.06 (t, *J* = 8.5 Hz, 2H), 6.35 (d, *J* =16.0 Hz, 1H), 7.36 (d, *J* =16.0 Hz, 1H), 7.58 (t, *J* = 7.5 Hz, 2H), 7.67 (t, *J* = 7.5 Hz, 1H), 7.74 (s, 1H), 8.00 (d, *J* = 7.5 Hz, 2H), 8.19 (s, 1H). ^{13}C-NMR (125MHz, DMSO-d_6): δ 24.47, 48.79, 118.60, 125.57, 125.64, 127.59, 129.16, 130.70, 133.76, 134.85, 137.49, 147.10, 156.13, 162.56. MS (ESI) *m*/*z*: 368.0 ([M+Na] $^{+}$). HRMS (ESI) for $C_{16}H_{15}N_3O_4S$ ([M-H]$^{-}$): calcd, 344.0705; found, 344.0686.

(*E*)-*N*-Hydroxy-3-(1-(3-methoxyphenylsulfonyl)-2,3-dihydro-1H-pyrrolo[2,3-b]pyridin-5-yl)acrylamide (9). The title compound was obtained in 40.80% overall yield

from compound **25c** in a similar manner as described for the preparation of **6** with m.p. 219.1-220.4 ^{o}C. ^{1}H-NMR (500 MHz, DMSO-d_6): δ 3.07 (t, *J* = 8.5 Hz, 2H), 3.79 (s, 3H), 4.03 (t, *J* = 8.5 Hz, 2H), 6.35 (d, *J* = 16.0 Hz, 1H), 7.22-7.24 (m, 1H), 7.37 (d, *J* = 16.0 Hz, 1H), 7.47-7.56 (m, 3H), 7.74 (s, 1H), 8.23 (s, 1H). ^{13}C-NMR (125MHz, DMSO-d_6): δ 24.47, 48.82, 55.61, 112.51, 118.67, 119.57, 119.75, 125.66, 125.78, 130.38, 130.76, 134.84, 138.45, 147.11, 156.12, 159.22, 162.55. MS (ESI) *m*/*z*: 398.0 ([M+Na]$^{+}$). HRMS (ESI) for $C_{17}H_{16}N_3O_5S$ ([M-H]$^{-}$): calcd, 374.0816; found, 374.0825.

(*E*)-N-Hydroxy-3-(1-(3-nitrophenylsulfonyl)-2,3-dihydro-1H-pyrrolo[2,3-b]pyridin-5-yl)acrylamide (10). The title compound was obtained in 71.16% overall yield from compound **25d** in a similar manner as described for the preparation of **6** with m.p. 158.8-160.3 ^{o}C. ^{1}H-NMR (500 MHz, CD_3OD+ DMSO-d_6): δ 3.14 (t, *J* = 8.5 Hz, 2H), 4.17 (t, *J* = 8.5 Hz, 2H), 6.39 (d, *J* = 16.0 Hz, 1H), 7.47 (d, *J* = 15.5 Hz, 1H), 7.75 (s, 1H), 7.84 (t, *J* = 8.0 Hz, 1H), 8.20 (s, 1H), 8.46 (d, *J* = 8.0 Hz, 1H), 8.48-8.50 (m, 1H), 8.90 (t, *J* = 9.0 Hz, 1H). ^{13}C-NMR (125MHz, DMSO-d_6): δ 24.56, 48.94, 118.92, 122.69, 125.74, 126.07, 128.35, 131.06, 131.21, 133.56, 134.72, 138.79, 146.92, 147.71, 155.64, 162.48. MS (ESI) *m*/*z*: 413.0 ([M+Na]$^{+}$). HRMS (ESI) for $C_{16}H_{13}N_4O_6S$ ([M-H]$^{-}$): calcd, 389.0561; found, 389.0543.

(*E*)-N-Hydroxy-4-(4-(5-((*E*)-3-(hydroxyamino)-3-oxoprop-1-enyl)-2,3-dihydro-1H-pyrrolo[2,3-b]pyridin-1-ylsulfonyl)phenyl)but-2-enamide (11). The title compound was obtained in 15.49% overall yield from compound **25e** in a similar manner as described for the preparation of **6** with m.p. 194.9-196.0 ^{o}C (decomp.). ^{1}H-NMR (500 MHz, DMSO-d$_6$): δ 3.07 (t, *J* = 8.5 Hz, 2H), 4.07 (t, *J* = 8.5 Hz, 2H), 6.34 (d, *J* = 16.0 Hz, 1H), 6.54 (d, *J* = 16.0 Hz, 1H), 7.36 (d, *J* = 16.0 Hz, 1H), 7.45 (d, *J* = 16.0 Hz, 1H), 7.72-7.84 (m, 3H), 8.00 (d, *J* = 8.5 Hz, 2H), 8.18 (s, 1H), 10.70 (s, 1H), 10.83 (s, 1H). ^{13}C-NMR (125MHz, DMSO-d$_6$): δ 24.50, 48.82, 118.65, 122.64, 125.63, 125.72, 127.94, 128.26, 130.74, 134.84, 136.38, 137.52, 139.92, 147.11, 156.05, 161.95, 162.55. MS (ESI) *m*/*z*: 453.0 ([M+Na]$^+$). HRMS (ESI) for $C_{19}H_{17}N_4O_6S$ ([M-H]$^-$): calcd, 429.0874; found, 429.0882.

(*E*)-N-Hydroxy-3-(3-(5-((*E*)-3-(hydroxyamino)-3-oxoprop-1-enyl)-2,3-dihydro-1H-pyrrolo[2,3-b]pyridin-1-ylsulfonyl)phenyl)acrylamide (12). The title compound was obtained in 37.17% overall yield from compound **25f** in a similar manner as described for the preparation of **6** with m.p. 219.6-220.7 ^{o}C. ^{1}H-NMR (500 MHz, DMSO-d$_6$): δ 3.09 (t, *J* = 8.5 Hz, 2H), 4.10 (t, *J* = 8.5 Hz, 2H), 6.35 (d, *J* = 16.0 Hz, 1H), 6.56 (d, *J* = 16.0 Hz, 1H), 7.36 (d, *J* = 16.0 Hz, 1H), 7.50 (d, *J* = 16.0 Hz, 1H), 7.61 (t, *J* = 8.5 Hz, 1H), 7.75 (s, 1H), 7.84 (d, *J* = 8.0 Hz, 1H), 7.97 (d, *J* = 8.0 Hz, 1H), 8.22 (d, *J* = 10.0 Hz, 2H), 10.71 (s, 1H), 10.84 (s, 1H). ^{13}C-NMR (125MHz,

DMSO-d_6): δ 24.51, 48.86, 118.69, 121.42, 125.67, 125.76, 125.81, 128.01, 129.89, 130.74, 132.86, 134.82, 135.83, 136.52, 138.13, 147.10, 156.04, 162.03, 162.55. MS (ESI) *m*/*z*: 453.0 ($[M+Na]^+$). HRMS (ESI) for $C_{19}H_{17}N_4O_6S$ ($[M-H]^-$): calcd, 429.0874; found, 429.0891.

(*E*)-*N*-Hydroxy-3-(2-(5-((*E*)-3-(hydroxyamino)-3-oxoprop-1-enyl)-2,3-dihydro-1H-pyrrolo[2,3-b]pyridin-1-ylsulfonyl)phenyl)acrylamide (13). The title compound was obtained in 33.19% overall yield from compound **25g** in a similar manner as described for the preparation of **6** with m.p. 220.2-221.4 ^{o}C. ^{1}H-NMR (500 MHz, DMSO-*d*$_6$): δ 3.10 (t, *J* = 8.5 Hz, 2H), 4.28 (t, *J* = 8.5 Hz, 2H), 6.26-6.33 (m, 2H), 7.31 (d, *J* = 16.0 Hz, 1H), 7.60-7.71 (m, 3H), 7.74 (s, 1H), 7.96 (s, 1H), 8.14-8.17 (m, 2H), 8.99 (br, 1H), 9.17 (br, 1H), 10.69 (s, 1H), 10.83 (s, 1H). ^{13}C-NMR (125MHz, DMSO-d_6): δ 24.37, 47.89, 118.40, 123.64, 125.12, 125.39, 128.04, 129.10, 130.45, 130.72, 133.86, 134.08, 134.30, 134.79, 137.08, 146.94, 155.65, 161.69, 162.49. MS (ESI) *m*/*z*: 453.0 ($[M+Na]^+$). HRMS (ESI) for $C_{19}H_{17}N_4O_6S$ ($[M-H]^-$): calcd, 429.0874; found, 429.0882.

5-Bromo-1-(4-methoxyphenylsulfonyl)-1H-pyrrolo[2,3-b]pyridine (27a). A mixture of 5-bromo-7-azaindole (**26**, 0.35 g, 1.78mmol) was dissolved in DMF (2mL) and added the 60% NaH (0.06 g, 2.67mmol) and stirred for a while. Then added the 4-methoxybenzenesulfonyl chloride (0.55 g, 2.67mmol) and stirred for overnight at

room temperature. The reaction was added H_2O to produce the precipitant. The residue was without more purification to afford **27a** (0.57 g, 87.20%) as a white solid. ^{1}H-NMR (500MHz, $CDCl_3$): δ 3.83 (s, 3H), 6.52 (d, *J* = 4.0 Hz, 1H), 6.93 (t, *J* = 9.0 Hz, 2H), 7.73 (t, *J* = 4.0 Hz, 1H), 7.96 (d, *J* = 2.0 Hz, 1H), 8.11 (d, *J* = 9.0Hz, 2H), 8.44 (d, *J* = 2.5 Hz, 1H).

5-Bromo-1-(4-fluorophenylsulfonyl)-1H-pyrrolo[2,3-b]pyridine (27b). The title compound was obtained in 93.32% overall yield from compound **26** in a similar manner as described for the preparation of **27a**. ^{1}H-NMR (500MHz, $CDCl_3$): δ 6.55 (d, *J* = 4.0 Hz, 1H), 7.17 (t, *J* = 9.0 Hz, 2H), 7.72 (t, *J* = 4.0 Hz, 1H), 7.99 (d, *J* = 2.5 Hz, 1H), 8.21-8.23 (m, 2H), 8.44 (d, *J* = 2.0 Hz, 1H).

5-bromo-1-(4-bromophenylsulfonyl)-1H-pyrrolo[2,3-b]pyridine (27c). The title compound was obtained in 81.20% overall yield from compound **26** in a similar manner as described for the preparation of **27a**. ^{1}H-NMR (500MHz, $CDCl_3$): δ 6.56 (d, *J* = 4.0 Hz, 1H), 7.64 (t, *J* = 9.0 Hz, 2H), 7.71 (t, *J* = 4.0 Hz, 1H), 7.99 (s, 1H), 8.04 (d, *J* = 9.0 Hz, 2H), 8.44 (d, *J* = 2.0 Hz, 1H).

5-Bromo-1-(3-bromophenylsulfonyl)-1H-pyrrolo[2,3-b]pyridine (27d). The title compound was obtained in 85.26% overall yield from compound **26** in a similar manner as described for the preparation of **27a**. ^{1}H-NMR (500MHz, $CDCl_3$): δ 6.57

(d, *J* = 3.5 Hz, 1H), 7.38 (t, *J* = 8.0 Hz, 1H), 7.71 (d, *J* = 4.5 Hz, 2H), 7.99 (d, *J* = 2.0 Hz, 1H), 8.13 (d, *J* = 8.0 Hz, 1H), 8.33 (s, 1H), 8.46 (d, *J* = 2.0 Hz, 1H).

5-Bromo-1-(2-bromophenylsulfonyl)-1H-pyrrolo[2,3-b]pyridine (27e). The title compound was obtained in 89.32% overall yield from compound **26** in a similar manner as described for the preparation of **27a**. ^{1}H-NMR (500MHz, $CDCl_3$): δ 6.57 (d, *J* = 4.0 Hz, 1H), 7.44 (t, *J* = 8.0 Hz, 1H), 7.57 (t, *J* = 8.0 Hz, 1H), 7.63 (d, *J* = 8.0, 1H), 7.96 (d, *J* = 4.0 Hz, 1H), 7.99 (d, *J* = 2.0 Hz, 1H), 8.25 (d, *J* = 1.5 Hz, 1H), 8.61 (d, *J* = 8.0 Hz, 1H).

(*E*)-3-(1-(4-Methoxyphenylsulfonyl)-1H-pyrrolo[2,3-b]20xpressi-5-yl)acrylic acid (28a). The title compound was obtained in 88.17% overall yield from compound **27a** in a similar manner as described for the preparation of **21**. ^{1}H-NMR (500MHz, CD_3OD): δ 3.82 (s, 3H), 6.57 (d, *J* = 16.0 Hz, 1H), 6.73 (d, *J* = 4.0 Hz, 1H), 7.03 (t, *J* = 9.0 Hz, 2H), 7.76 (d, *J* = 16.0 Hz, 1H), 7.83 (t, *J* = 4.0 Hz, 1H), 8.08 (d, *J* = 9.0Hz, 2H), 8.24 (d, *J* = 2.0 Hz, 1H), 8.52 (d, *J* = 2 Hz, 1H).

(*E*)-3-(1-(4-Fluorophenylsulfonyl)-1H-pyrrolo[2,3-b] 20 xpressi-5-yl)acrylic acid (28b). The title compound was obtained in 63.12% overall yield from compound **27b** in a similar manner as described for the preparation of **21**. ^{1}H-NMR (500MHz, CD_3OD): δ 6.58 (d, *J* = 16.0 Hz, 1H), 6.77 (d, *J* = 3.5 Hz, 1H), 7.29 (t, *J* = 8.5 Hz,

2H), 7.76 (d, *J* = 16.0 Hz, 1H), 7.85 (t, *J* = 4.0 Hz, 1H), 8.22-8.25 (m, 2H), 8.26 (s, 1H), 8.53 (d, *J* = 2.0 Hz, 1H).

(*E*)-3-(4-(5-((*E*)-2-Carboxyvinyl)-1H-pyrrolo[2,3-b] 21 xpressi-1-ylsulfonyl)phenyl) acrylic acid (28c). The title compound was obtained in 88.31% overall yield from compound **27c** in a similar manner as described for the preparation of **21**. ^{1}H-NMR (500MHz, DMSO-d_6): δ 6.63 (d, *J* = 16.0 Hz, 1H), 6.65 (d, *J* = 16.0 Hz, 1H), 6.85 (d, *J* = 4.0 Hz, 1H), 7.57 (d, *J* = 15.5 Hz, 1H), 7.68 (d, *J* = 16.0 Hz, 1H), 7.91 (d, *J* = 8.5 Hz, 2H), 7.96 (d, *J* = 4.0 Hz, 1H), 8.10 (d, *J* = 8.5 Hz, 2H), 8.41 (d, *J* = 2.0 Hz, 1H), 8.67 (d, *J* = 1.5 Hz, 1H).

(*E*)-3-(3-(5-((*E*)-2-Carboxyvinyl)-1H-pyrrolo[2,3-b] 21 xpressi-1-ylsulfonyl)phenyl) acrylic acid (28d). The title compound was obtained in 87.57% overall yield from compound **27d** in a similar manner as described for the preparation of **21**. ^{1}H-NMR (500MHz, CD_3OD+ DMSO-d_6): δ 6.62 (d, *J* = 16.0 Hz, 2H), 6.83 (d, *J* = 4.0 Hz, 1H), 7.63 (d, *J* = 8.0 Hz, 1H), 7.69 (d, *J* = 16.0 Hz, 1H), 7.77 (d, *J* = 16.0 Hz, 1H), 7.94 (d, *J* = 4.0 Hz, 2H), 8.18 (d, *J* = 8.0 Hz, 1H), 8.31 (d, *J* = 2.0 Hz, 1H), 8.42 (s, 1H), 8.60 (d, *J* = 2.0 Hz, 1H).

(*E*)-3-(2-(5-((*E*)-2-Carboxyvinyl)-1H-pyrrolo[2,3-b] 21 xpressi-1-ylsulfonyl)phenyl) acrylic acid (28e). The title compound was obtained in 81.24% overall yield from compound **27e** in a similar manner as described for the preparation of **21**. ^{1}H-NMR

(500MHz, CD_3OD+DMSO-d_6): δ 6.15 (d, *J* = 16.0 Hz, 1H), 6.55 (d, *J* = 16.0 Hz, 1H), 6.76 (d, *J* = 4.0 Hz, 1H), 7.66-7.74 (m, 4H), 7.91 (d, *J* = 4.0 Hz, 1H), 8.26 (s, 1H), 8.39-8.42 (m, 2H), 8.45 (d, *J* = 16.0 Hz, 1H).

(*E*)-N-Hydroxy-3-(1-(4-methoxyphenylsulfonyl)-1H-pyrrolo[2,3-b]pyridin-5-yl) acrylamide (14). The title compound was obtained in 90.91% overall yield from compound **28a** in a similar manner as described for the preparation of **6** with m.p. 192.5-193.3. ^{1}H-NMR (500MHz, DMSO-d_6): δ 3.78 (s, 3H), 6.52 (d, *J* = 16.0 Hz, 1H), 6.81 (d, *J* = 4.0 Hz, 1H), 7.10 (t, *J* = 9.0 Hz, 2H), 7.54 (d, *J* = 16.0 Hz, 1H), 7.89 (t, *J* = 4.0 Hz, 1H), 8.03 (d, *J* = 9.0Hz, 2H), 8.23 (d, *J* = 1.5 Hz, 1H), 8.55 (d, *J* = 1.5 Hz, 1H). ^{13}C-NMR (125MHz, DMSO-d_6): δ 55.85, 105.88, 114.77, 119.78, 122.57, 126.54, 127.82, 127.91, 128.64, 130.01, 135.45, 144.75, 146.67, 162.44, 163.93. HRMS (ESI) for $C_{17}H_{16}N_3O_5S$ ([M+H]$^+$): calcd, 374.0811; found, 374.0822.

(*E*)-3-(1-(4-Fluorophenylsulfonyl)-1H-pyrrolo[2,3-b]pyridin-5-yl)-N-hydroxy-acrylamide (15). The title compound was obtained in 86.69% overall yield from compound **28b** in a similar manner as described for the preparation of **6** with m.p. 213.6-214.5. ^{1}H-NMR (500MHz, DMSO-d_6): δ 6.52 (d, *J* = 15.5 Hz, 1H), 6.86 (d, *J* = 4.0 Hz, 1H), 7.47 (t, *J* = 9.0 Hz, 2H), 7.55 (d, *J* = 15.5 Hz, 1H), 7.93(d, *J* = 4.0 Hz, 1H), 8.18-8.21 (m, 2H), 8.25 (s, 1H), 8.56 (s, 1H). ^{13}C-NMR (125MHz, DMSO-d_6): δ 106.42, 116.92, 117.11, 119.98, 122.68, 126.82, 127.88, 128.03, 130.91, 130.99,

133.63, 135.37, 144.88, 146.70, 162.41, 164.44, 166.46. HRMS (ESI) for $C_{16}H_{13}FN_3O_4S$ ($[M+H]^+$): calcd, 362.0611; found, 362.0618.

(*E*)-N-Hydroxy-3-(4-(5-((*E*)-3-(hydroxyamino)-3-oxoprop-1-enyl)-1H-pyrrolo[2,3-b]pyridin-1-ylsulfonyl)phenyl)acrylamide (16). The title compound was obtained in 38.90% overall yield from compound **28c** in a similar manner as described for the preparation of **6** with m.p. 188.7-190.5 (decomp.). ^{1}H-NMR (500MHz, DMSO-d_6): δ 6.54 (t, *J* = 16.0 Hz, 2H), 6.86 (d, *J* = 4.0 Hz, 1H), 7.44 (d, *J* = 15.5 Hz, 1H), 7.54 (d, *J* = 15.5 Hz, 1H), 7.77 (d, *J* = 8.5 Hz, 2H), 7.93 (d, *J* = 4.0 Hz, 1H), 8.10 (d, *J* = 8.5 Hz, 2H), 8.25(s, 1H), 8.56(s, 1H). ^{13}C-NMR (125MHz, DMSO-d_6): δ 106.43, 119.96, 122.67, 123.36, 126.80, 127.94, 128.15, 128.35, 135.34, 136.06, 137.17, 140.95, 144.91, 146.76, 162.38. HRMS (ESI) for $C_{19}H_{17}N_4O_6S$ ($[M+H]^+$): calcd, 429.0869; found, 429.0876.

(*E*)-N-Hydroxy-3-(3-(5-((*E*)-3-(hydroxyamino)-3-oxoprop-1-enyl)-1H-pyrrolo[2,3-b]pyridin-1-ylsulfonyl)phenyl)acrylamide (17). The title compound was obtained in 67.43% overall yield from compound **28d** in a similar manner as described for the preparation of **6** with m.p. 219.4-221.3 (decomp.). ^{1}H-NMR (500MHz, DMSO-d_6): δ 6.52 (t, *J* = 15.5 Hz, 1H), 6.59 (d, *J* = 16.0 Hz, 1H), 6.87 (d, *J* = 4.0 Hz, 1H), 7.50 (d, *J* = 16.0 Hz, 1H), 7.55 (d, *J* = 15.5 Hz, 1H), 7.64 (t, *J* = 8.5 Hz, 1H), 7.89 (d, *J* = 8.0 Hz, 1H), 7.94 (d, *J* = 4.0 Hz, 1H), 8.05 (d, *J* = 8.0 Hz, 1H), 8.25(s, 1H), 8.34 (s, 1H),

8.59(s, 1H). ^{13}C-NMR (125MHz, DMSO-d_6): δ 106.44, 119.99, 121.98, 122.68, 125.67, 126.84, 127.76, 127.92, 128.03, 130.39, 133.74, 135.34, 136.10, 136.30, 138.08, 144.89, 146.72, 161.93, 162.39. HRMS (ESI) for $C_{19}H_{17}N_4O_6S$ ([M+H]$^+$): calcd, 429.0869; found, 429.0884.

(*E*)-N-Hydroxy-3-(2-(5-((*E*)-3-(hydroxyamino)-3-oxoprop-1-enyl)-1H-pyrrolo[2,3-b]pyridin-1-ylsulfonyl)phenyl)acrylamide (18). The title compound was obtained in 67.20% overall yield from compound **28e** in a similar manner as described for the preparation of **6** with m.p. 159.6-161.9 (decomp.). ^{1}H-NMR (500MHz, DMSO-d_6): δ 6.19 (d, *J* = 15.5 Hz, 1H), 6.49 (d, *J* = 5.5 Hz, 1H), 6.84 (d, *J* = 4.0 Hz, 1H), 7.51 (d, *J* = 16.0 Hz, 1H), 7.65-7.70 (m, 2H), 7.76 (t, *J* = 7.0 Hz, 1H), 7.86 (d, *J* = 4.0 Hz, 1H), 8.10 (d, *J* = 15.5 Hz, 1H), 8.24 (s, 1H), 8.27 (d, *J* = 8.0 Hz, 1H), 8.41 (s, 1H). ^{13}C-NMR (125MHz, DMSO-d_6): δ 105.63, 119.94, 122.46, 124.86, 126.80, 127.68, 127.94, 128.68, 129.48, 130.91, 133.08, 134.56, 135.25, 135.33, 144.84, 146.55, 161.34, 162.40. HRMS (ESI) for $C_{19}H_{16}NaN_4O_6S$ ([M+Na]$^+$): calcd, 451.0688; found, 451.0683.
